# Electrical Manipulation of Field‐Free Magnetization Switching Driven by Spin‐Orbit Torque in Amorphous Gradient‐Mn_3_Sn

**DOI:** 10.1002/advs.202417621

**Published:** 2025-03-24

**Authors:** Mingfang Zhang, Bin Cui, Taiyu An, Xue Ren, Weikang Liu, Xiangxiang Zhao, Hehe Ding, Zhiyu Zhang, Xu Zhang, Weijie Kuai, Guangjun Zhou, Bin Cheng, Liang Liu, Jifan Hu

**Affiliations:** ^1^ School of Physics State Key Laboratory for Crystal Materials Shandong University Jinan 250100 China; ^2^ School of Integrated Circuits Shandong University Jinan 250100 China

**Keywords:** amorphous gradient‐Mn_3_Sn, electrical manipulation, field‐free magnetization switching, hydrogen migration, spin‐orbit torque

## Abstract

Switching the magnetization without an assisted magnetic field is crucial for the application of spin‐orbit torque (SOT) devices. However, the realization of field‐free magnetization switching usually calls for intricate design and growth of heterostructure. In this study, it is found that the amorphous Mn_3_Sn can generate a highly efficient spin current with a strong *z*‐direction polarization component due to its spontaneous composition gradient, which switches the perpendicular magnetization in the absence of an external field. The SOT efficiency of gradient‐Mn_3_Sn can be reversibly modulated by the ionic liquid gating based on the migration of hydrogen ions, which reverses the polarity of field‐free magnetization switching and allows the realization of 16 binary Boolean logic functions in a single device by pure electrical methods. These results not only offer a very convenient route to field‐free magnetization switching but also can promote the development of in‐memory computing for spintronic devices.

## Graphical abstract

A highly efficient spin current with *z*‐polarization is achieved in amorphous Mn_3_Sn with a spontaneous composition gradient, which allows field‐free magnetization switching. Spin current efficiency and magnetization switching polarity are reversibly manipulated by ionic liquid gating, enabling logic functions by pure electrical methods. This work offers a very convenient route to in‐memory computing in spintronic devices with low energy dissipation.

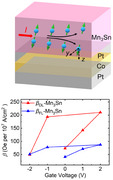

## 1 Introduction

The spin‐orbit torque (SOT) derived from the spin Hall effect (SHE) or Rashba‐Edelstein effect (REE) has been shown to enable the electrical switching of magnetization.^[^ ^1^, ^2^, ^3^, ^4^ ^]^ In most cases, an external magnetic field is required to break mirror symmetry and achieve SOT‐induced magnetization switching in a perpendicular magnetic anisotropy (PMA) system.^[^ ^5^, ^6^ ^]^ The field‐free magnetization switching is widely pursued due to its advantage in low power consumption and convenience for practical application.^[^ ^7^, ^8^, ^9^, ^10^ ^]^ Recently, a large number of methods have been employed to obtain field‐free magnetization switching such as introducing an in‐plane effective field through adjacent (anti‐)ferromagnetic coupling^[^ ^11^, ^12^, ^13^, ^14^ ^]^ or Dzyaloshinskii‐Moriya interaction (DMI),^[^ ^5^, ^6^, ^15^ ^]^ breaking mirror symmetry by a wedge structure,^[^ ^16^, ^17^ ^]^ creating an effective out‐of‐plane field based on competing spin currents,^[^ ^18^ ^]^ using spin current with out‐of‐plane spin polarization,^[^ ^8^, ^19^, ^20^ ^]^ or the lateral SOTs.^[^ ^21^, ^22^ ^]^ However, the complex structures or stringent growth conditions limit the practical application of these methods.

Mn_3_Sn is a classic noncollinear antiferromagnetic material, which exhibits exotic properties such as large anomalous Hall effect and Weyl semimetal phase.^[^ ^23^, ^24^ ^]^ Furthermore, the crystalline Mn_3_Sn (0001) thin film has been proven to be able to generate spin current with *z*‐direction polarization (*σ_z_*),^[^ ^25^, ^26^, ^27^ ^]^ supplying a novel way to the realization of field‐free magnetization switching. However, the generation of *σ_z_* spin current in Mn_3_Sn is still under intense debate, with several mechanisms proposed to date, such as the magnetic spin Hall effect^[^ ^28^, ^29^ ^]^ and the interfacial scattering effect.^[^ ^25^ ^]^ On the other hand, the preparation of crystalline Mn_3_Sn calls for the accuracy control of Mn‐Sn composition and annealing temperature,^[^ ^30^ ^]^ especially in consideration of the ferromagnetic Mn_2_Sn phase with lower formation energy.^[^ ^31^ ^]^ Thus, a more convenient and feasible acquisition of *σ_z_* spin current in Mn_3_Sn with a clear mechanism is crucial to make it a realistic candidate for SOT devices.

Besides the field‐free switching of magnetization by SOT, the polarity of magnetization switching is also an interesting topic.^[^ ^32^, ^33^ ^]^ Usually, the polarity of the magnetization switching is determined by the directions of the external magnetic field and spin current polarization. Nevertheless, once the switching polarity can be reversed back and forced with lower energy consumption, for example by an electric field, it can be used to realize full‐electrically controlled spin logic operations in a single device. In this work, we demonstrate that a sizeable *σ_z_* spin current is generated by an amorphous Mn_3_Sn layer with a spontaneous composition gradient, which drives the switching of magnetization without an external field. Such a *σ_z_* spin current is successfully manipulated through ionic liquid (IL) gating due to the reversible injection and extraction of hydrogen ions. Moreover, the IL gating could reverse the polarity of SOT‐driven magnetization switching, which allows the realization of all 16 Boolean logic operations by pure electrical methods. Our results supply a feasible way to realize and manipulate *σ_z_* spin current in Mn_3_Sn, which makes it straightforward for the practical application of in‐memory computing spintronic devices.

## 2 Results and Discussion

### 2.1 Field‐Free SOT‐Induced Magnetization Switching in Mn_3_Sn‐Based Heterostructure

**Figure 1:**
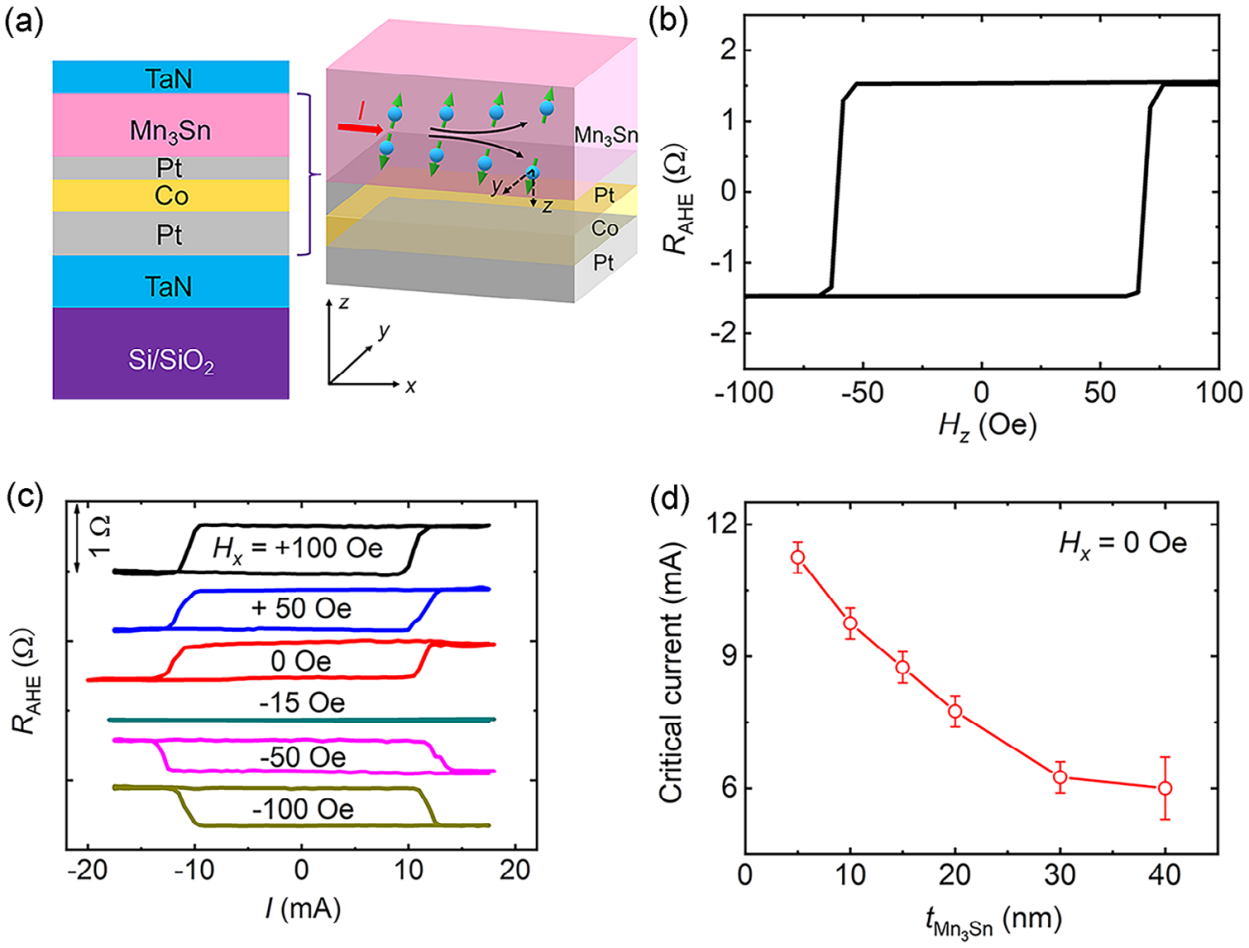
SOT‐induced magnetization switching of TaN/Pt/Co/Pt/Mn_3_Sn/TaN heterostructure. a) Diagram of the TaN/Pt/Co/Pt/Mn_3_Sn/TaN heterostructure. The charge current (*I*) is applied along the *x*‐direction, the Hall voltage is measured along the *y*‐direction, and the *z*‐direction is perpendicular to the thin film or device plane. The spin current in Mn_3_Sn has both *σ_y_* and *σ_z_* polarizations. b) Dependence of *R*_AHE_ on *H_z_* for TaN(5)/Pt(1.5)/Co(0.9)/Pt(1)/Mn_3_Sn(5)/TaN(1). c) SOT‐induced magnetization switching with different *H_x_*. d) Dependence of the critical current on *t*_Mn3Sn_ with *H_x_* = 0 Oe.

A series of TaN(5)/Pt(*t*_Pt_)/Co(0.9)/Pt(1)/Mn_3_Sn(*t*_Mn3Sn_)/TaN(1) heterostructures (units in nanometer) with different Pt and Mn_3_Sn thicknesses (*t*_Pt_ and *t*_Mn3Sn_) are deposited on Si/SiO_2_ substrate by magnetron sputtering at room temperature and then fabricated into Hall bar devices for transport measurements (**Figure** **1**a). Here the Mn_3_Sn layer is amorphous (Figure S1, Supporting Information) with weak magnetization (Figure S2, Supporting Information). Figure 1b shows the anomalous Hall resistance (*R*_AHE_) of TaN(5)/Pt(1.5)/Co(0.9)/Pt(1)/Mn_3_Sn(5)/ TaN(1) device, where the sample exhibits good PMA. The SOT‐induced magnetization switching measurement is then performed with different in‐plane magnetic fields *H_x_* varying from +100 to −100 Oe as shown by *R*_AHE_‐*I* loops in Figure 1c. Remarkably, the SOT‐induced magnetization switching without an external in‐plane magnetic field (*H_x_* = 0) is successfully achieved and the critical current is ≈11.5 mA. The polarity of magnetization switching is anticlockwise when *H_x_* is positive or 0, while the polarity turns clockwise once a large negative *H_x_* is applied. It is noteworthy that the SOT‐induced magnetization switching has vanished at *H_x_* = −15 Oe, indicating that an in‐plane effective field of ≈+15 Oe has existed in the sample.^[^ ^15^ ^]^ Different from the sample with the Mn_3_Sn layer, the SOT‐induced magnetization switching cannot be realized in Pt/Co/Pt control samples without an external field (Figure S3, Supporting Information). While the field‐free switching can still be achieved in TaN(5)/Pt(1)/Co(0.9)/Pt(1)/Mn_3_Sn(5)/TaN(1) where two Pt layers have the same thickness, suggesting that the Mn_3_Sn layer, rather than asymmetric Pt layers, is crucial for the field‐free magnetization switching (Figure S4, Supporting Information).

In consequence, we turn to investigate the role of the Mn_3_Sn layer in the field‐free SOT‐induced magnetization switching by changing the *t*_Mn3Sn_ from 5 to 40 nm. No matter what the thickness of the Mn_3_Sn layer is, the magnetization can always be reversed by the pulsed current without the application of an external magnetic field (Figure S5, Supporting Information), and the dependence of critical current for magnetization switching on *t*_Mn3Sn_ is summarized in Figure 1d. With the increase of Mn_3_Sn layer thickness from 5 to 40 nm, the critical current gradually decreases from 11.5 to 6.0 mA, suggesting that the SOT in Mn_3_Sn originated from the bulk SHE rather than the interfacial REE. The spin currents mainly originate from the Mn_3_Sn layer in these samples. As the thickness of the Mn_3_Sn layer gradually increases, the critical current initially decreases and eventually stabilizes, reaching its diffusion length limit at 40 nm. In addition, according to the polarity of magnetization switching, the polarization (in‐plane component) of spin current generated by the Mn_3_Sn layer is opposite to that from the Pt layer (Figure S6, Supporting Information).^[^ ^34^ ^]^

**Figure 2:**
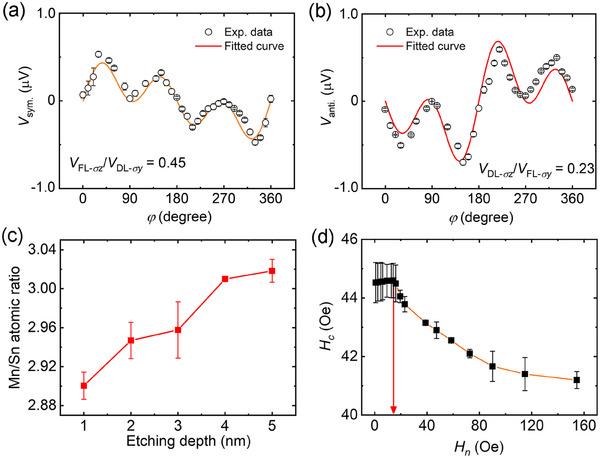
Mechanism of the field‐free SOT‐induced magnetization switching. Angle‐dependent symmetric and antisymmetric ST‐FMR signals of Ni_81_Fe_19_(15)/Mn_3_Sn(20) at 4 GHz: a) *V*_sym._−*φ* curve and b) *V*_anti._ −*φ* curve. The *φ* is the in‐plane angle between the current and magnetic field. Open circles with error bars are the experimental data and the solid curves represent the fitted results. c) Dependence of Mn/Sn atomic ratio on the etching depth for Mn_3_Sn layer in TaN(5)/Pt(1.5)/Co(0.9)/Pt(1)/Mn_3_Sn(5)/TaN(1). The etching depth of 1 nm corresponds to the surface, while that of 5 nm corresponds to the bottom. d) The coercive field *H*_c_ as a function of the *H*_n_ in TaN(5)/Pt(1.5)/Co(0.9)/Pt(1)/Mn_3_Sn(5)/TaN(1). The red arrow indicates the DMI field of ≈14 Oe.

In order to study the reason for field‐free magnetization switching caused by Mn_3_Sn, the polarization direction of spin current is characterized by the angle‐dependent spin‐torque ferromagnetic resonance (ST‐FMR) technique. Here a Ni_81_Fe_19_(15)/Mn_3_Sn(20) sample is used for ST‐FMR measurement because the Ni_81_Fe_19_ with in‐plane magnetization anisotropy can enhance the spin‐torque related signal generated by Mn_3_Sn. The symmetric and antisymmetric components of ST‐FMR results (*V*_sym._ and *V*_anti._) are separated and displayed as a function of angle *φ* (*φ* is the in‐plane angle between the magnetic field and current) in **Figure** **2**a, b, respectively (see raw ST‐FMR data in Figure S7, Supporting Information). The contribution of spin current with in‐plane polarization (*σ_y_*) and out‐of‐plane polarization (*σ_z_*) could be separated as follows:^[^ ^20^, ^35^ ^]^

(1) $$V_{\mathrm{sym}.\left(\mathrm{anti}.\right)}=V_{\sigma y}\sin2\varphi \cdot \cos\varphi +V_{\sigma z}\sin2\varphi$$

where *V_σy_* and *V_σz_* are the voltages produced by *σ_y_*‐ and *σ_z_*‐induced spin torques. The *V*_sym._ reflects the damping‐like torque (*τ*_DL_) from *σ_y_* spin current (*V*_DL‐_ *_σy_*) and the field‐like torque (*τ*_FL_) from *σ_z_* spin current (*V*_FL‐_ *_σz_*), while the *V*_anti._ contains the information of *τ*_FL_ from *σ_y_* spin current (*V*_FL‐_ *_σy_*) and *τ*_DL_ from *σ_z_* spin current (*V*_DL‐_ *_σz_*). By fitting the *V*_sym._ and *V*_anti._ based on Equation (1), the voltage ratios of *V_σz_* and *V_σy_* are |*V*_FL‐_ *_σz_*/*V*_DL‐_ *_σy_* | = 0.45 and |*V*_DL‐_ *_σz_*/*V*_FL‐_ *_σy_* | = 0.23, which demonstrates that a sizable *σ_z_* exists in the Ni_81_Fe_19_(15)/Mn_3_Sn(20). In sharp contrast to Ni_81_Fe_19_(15)/Mn_3_Sn(20) sample, the *V_σz_*‐related signals in Ni_81_Fe_19_(10)/Pt(10) sample are almost negligible (Figure S8, Supporting Information), confirming that the *σ_z_* spin current comes from the Mn_3_Sn layer.^[^ ^36^ ^]^

It should be noted that although the *σ_z_* spin current has been observed in crystalline Mn_3_Sn because of the chiral antiferromagnetic structure,^[^ ^23^, ^24^ ^]^ such a mechanism is not applicable in our amorphous Mn_3_Sn. In order to clarify the origin of *σ_z_* spin current in the Mn_3_Sn, the depth‐resolved X‐ray photoelectron spectra (XPS) are conducted to study the compositions of Mn and Sn elements as shown in Figure 2c. Interestingly, the composition Mn_3_Sn layer is not uniform and the atomic ratios of Mn/Sn gradually increase from 2.90 at the surface to 3.02 at the bottom. Such a composition gradient could be eliminated, increased, or even reversed artificially by changing the power of the film growth (more details in Experimental Section), which dramatically changes the magnetization switching behavior (Figures S9,S10, Supporting Information). Although the exact reason why the gradient can be formed in Mn_3_Sn is still not clear, the Pt layer should play a critical role (Figure S11, Supporting Information). One possible reason is that the Pt‐Mn alloy is easier to form at the interface due to their relatively closer melting points (*T_M_*_‐Pt_ = 1772 °C, *T_M_*_‐Mn_ = 1244 °C, *T_M_*_‐Sn_ = 232 °C).

To further investigate the mechanism of how composition gradient induces *σ_z_* spin current, we turn to study the gradient‐related DMI field (*H*_DMI_) in the heterostructure. A method based on the magnetic droplet nucleation models used to quantify the *H*_DMI_ in TaN(5)/Pt(1.5)/Co(0.9)/Pt(1)/Mn_3_Sn(5)/TaN(1) (see Figure S12, Supporting Information),^[^ ^37^, ^38^ ^]^ and Figure 2d shows the coercive field *H*_c_ as a function of accompanying in‐plane field *H*_n_. In this manner, *H*_DMI_ is determined to be ≈14 Oe, which is close to the effective in‐plane field of 15 Oe obtained in Figure 1c. Such an *H*_DMI_ is robust and reconfirmed in samples with thicker Mn_3_Sn (Figures S13,S14, Supporting Information). According to a previous report,^[^ ^39^ ^]^ the *σ_z_* spin current is generated when the carrier spins are rotated by the spin‐orbit field *H*_SO_: σ_*z*_ ∝ *H*_SO_  ×  *T*, where *T* is the magnetic moment. Here we think that the *H*_DMI_ plays a similar role as *H*_SO_, and the *σ_z_* spin current is the cross‐product of in‐plane *H*_DMI_ and the magnetic moment of Mn (the magnetic moment of Mn has prominent in‐plane component as demonstrated by X‐ray magnetic circular dichroism (XMCD) results in Figure S15, Table S1, Supporting Information). It should be noted that the composition gradient‐driven DMI and field‐free switching have been reported in CoTb and CoPt systems,^[^ ^5^, ^6^ ^]^ which calls for precise control of growth conditions. The gradient‐Mn_3_Sn here is spontaneous, which makes the sample preparation much simpler and more convenient. Meanwhile, it is more favorable for most PMA systems because no thick ferromagnetic layer is needed to create the composition gradient.

### 2.2 Electrical Manipulation of the SOT Efficiency

**Figure 3:**
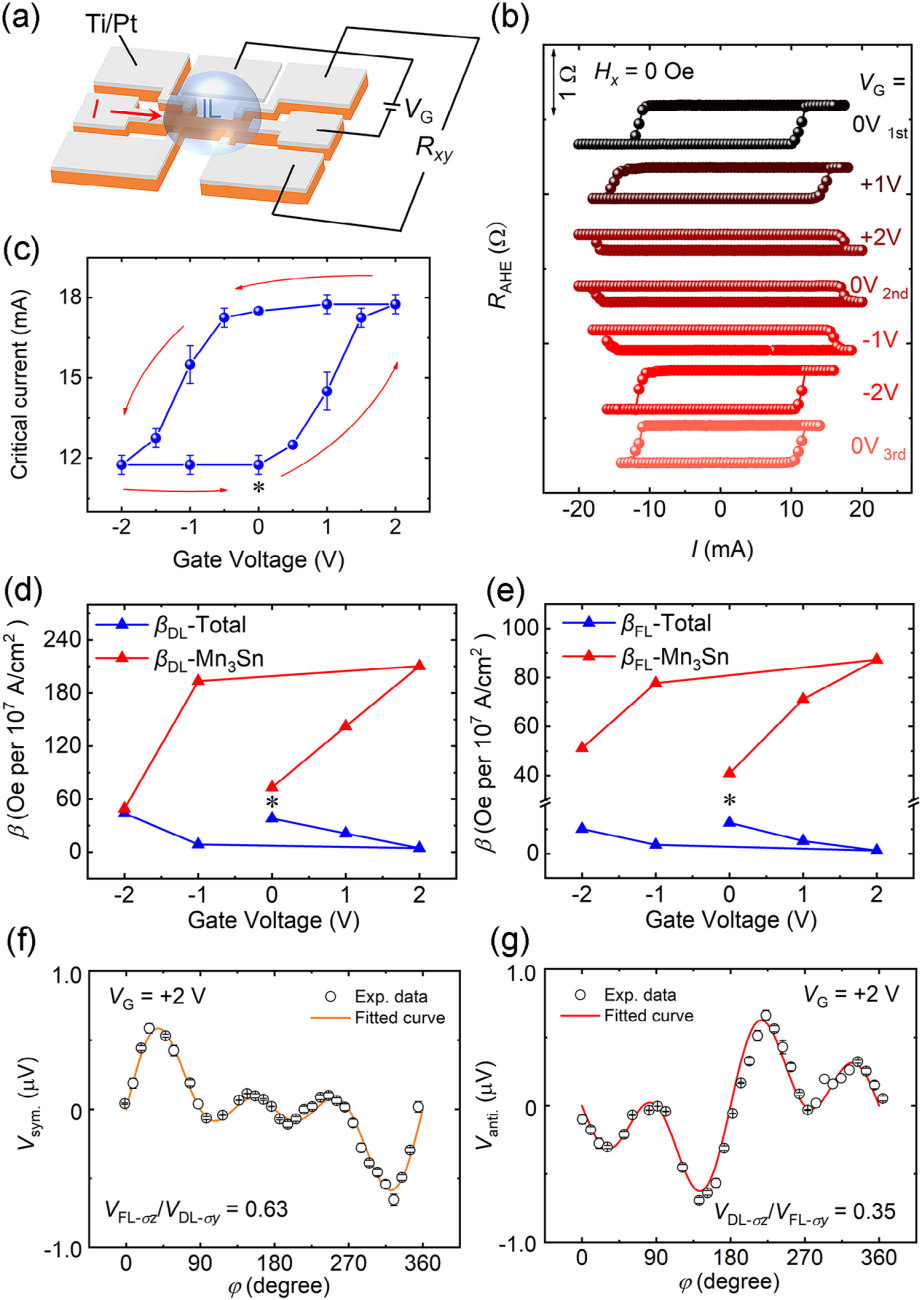
Manipulation of SOT generation efficiency through gate voltage. a) Schematic diagram of the device structure for transport measurement under different *V*_G_. b) *R*_AHE_ vs *I* loops for TaN(5)/Pt(1.5)/Co(0.9)/Pt(1)/Mn_3_Sn(5)/TaN(1) under different *V*_G_ with *H_x_* = 0 Oe. c) The evolution of the critical current under different *V*_G_, where the “*” represents the initial state before applying the *V*_G_. The gate voltage is applied in the sequence of 0 V_1st_ → +2 V → 0 V_2nd_ → −2 V → 0 V_3rd_, which is indicated by the red arrows. The SOT efficiency *β* for Mn_3_Sn and total device under different *V*_G_ d) *β*_DL_ and e) *β*_FL_. *V*_G_ is applied in the sequence of 0 V → +2 V → −2 V. *β*_DL/FL_ is defined as *H*_DL/FL_/*J*, where *J* is the current density. Angle‐dependent symmetric and antisymmetric ST‐FMR signals of Ni_81_Fe_19_(15)/Mn_3_Sn(20) at 4 GHz with *V*_G_ = +2 V: a) *V*_sym._−*φ* curve and b) *V*_anti._ −*φ* curve. The *φ* is the in‐plane angle between the current and magnetic field. Open circles with error bars are the experimental data and the solid curves represent the fitted results.

The *σ_z_* spin current generated by the Mn_3_Sn layer results in an efficient field‐free magnetization switching. Besides that, we also provide a possibility to dynamically modulate the SOT and the polarity of SOT‐induced magnetization switching by electrical manipulation. **Figure** **3**a shows the device and transport measurement geometry, where various gate voltages (*V*_G_) are applied through the IL. Typical SOT‐induced magnetization switching results (*H_x_* = 0 Oe) for TaN(5)/Pt(1.5)/Co(0.9)/Pt(1)/Mn_3_Sn(5)/TaN(1) under different *V*_G_ are displayed in Figure 3b. Although SOT‐induced field‐free magnetization switching is achieved in the *V*_G_ range between −2 and +2 V, the magnitude of critical current is quite different as we summarized in Figure 3c. The critical current increases dramatically from 11.5 to 17.5 mA when *V*_G_ increases from 0 to +2 V. Such a manipulation of critical current is maintained even when *V*_G_ is gradually decreased to zero, exhibiting a non‐volatile character. On the contrary, the application of negative *V*_G_ induces a remarkable decrease in the critical current (11.5 mA at *V*_G_ = −2 V), suggesting that the IL gating effect is almost reversible. Another interesting phenomenon is that the switching polarity is reversed from initial anti‐clockwise to clockwise at *V*_G_ = +2 V, which suggests that the SOT‐induced switching is changed from Pt‐dominated to Mn_3_Sn‐dominated (the relationship between switching polarity and spin current polarization is discussed in Figure S16, Supporting Information).

Subsequently, the harmonic voltage measurements under different *V*_G_ are studied to investigate the effect of the electric field on SOT efficiency in depth. The dependences of the damping‐like and field‐like SOT efficiencies (*β*_DL_ and *β*_FL_) of TaN(5)/Pt(1.5)/Co(0.9)/Pt(1)/Mn_3_Sn(5)/TaN(1) (labeled by total) on the *V*_G_ are shown in Figure 3d, e, respectively (see raw data and analysis in Figure S17, Supporting Information).^[^ ^12^, ^40^, ^41^ ^]^ When the positive *V*_G_ is increased from 0 to +2 V, the SOT efficiencies of the total device $\beta_{\mathrm{Total}}^{\mathrm{DL}}$ and $\beta_{\mathrm{Total}}^{\mathrm{FL}}$ are reduced from 38.0 to 4.6 Oe per 10^7^A cm^−2^ and 12.6 to 1.3 Oe per 10^7^A cm^−2^, respectively. When the *V*_G_ increases in the negative direction to −2 V, the $\beta_{\mathrm{Total}}^{\mathrm{DL}}$ and $\beta_{\mathrm{Total}}^{\mathrm{FL}}$ are ≈43.9 and 10.1 Oe per 10^7^A cm^−2^, respectively, which are close to those initial values. The variation of *β*_DL/FL_ with *V*_G_ is well in line with the change of critical current magnitude in the magnetization switching (Figure 3c).

As the modulation effect of IL gating is usually limited to the surface layer (Mn_3_Sn layer), we extract the SOT efficiencies of the Mn_3_Sn layer ($\beta_{\mathrm{Mn}3\mathrm{Sn}}^{\mathrm{DL}}$ and $\beta_{\mathrm{Mn}3\mathrm{Sn}}^{\mathrm{FL}}$) at different *V*_G_ by deducting the SOT efficiencies of a TaN(5)/Pt(1.5)/Co(0.9)/Pt(1)/TaN(1) heterostructure based on a shunting model (see Table S2, Supporting Information). As shown in Figure 3d, e (labelled by Mn_3_Sn), the SOT efficiencies of Mn_3_Sn layer under *V*_G_ = +2 V are $\beta_{\mathrm{Mn}3\mathrm{Sn}}^{\mathrm{DL}}$ = 210.4 Oe per 10^7^A cm^−2^ and $\beta_{\mathrm{Mn}3\mathrm{Sn}}^{\mathrm{FL}}$ = 87.3 Oe per 10^7^A cm^−2^, which are much larger than the initial values of $\beta_{\mathrm{Mn}3\mathrm{Sn}}^{\mathrm{DL}}$ = 73.3 Oe per 10^7^A cm^−2^ and $\beta_{\mathrm{Mn}3\mathrm{Sn}}^{\mathrm{FL}}$ = 40.8 Oe per 10^7^A cm^−2^ at *V*_G_ = 0 V. On the contrary, a negative *V*_G_ = −2 V reduces the efficiencies almost back to the initial states. The SOT efficiencies of total heterostructure and Mn_3_Sn layer behave opposite responses to the gate voltage due to the inverse spin current polarizations (in‐plane component) of Pt and Mn_3_Sn, which could also be reflected by the reversal of switching polarity at *V*_G_ = +2 V (Figure 3b) and reconfirmed in the heterostructure with 10 nm‐thick Mn_3_Sn (Section S15, Supporting Information). It should be noted that the SOT efficiency in Mn_3_Sn is much bigger than that of previously reported Pt (*β*_DL_ = 17.0 Oe per 10^7^A cm^−2^ and *β*_FL_ = 13.0 Oe per 10^7^A cm^−2^),^[^ ^42^ ^]^ indicating that our amorphous Mn_3_Sn shows very good potential as a high‐efficient SOT source. Based on the shunting model, the critical switching current density in the gradient‐Mn_3_Sn layer is 17.4×10^5^ A cm^−2^ (*V*_G_ = 0 V), which is lower than most heavy metals. Despite the magnitude of SOT efficiency, the spin polarization direction is also changed by the gate voltage. We characterize the *σ_y_*‐ and *σ_z_*‐induced spin torques at *V*_G_ = +2 V by angle‐dependent ST‐FMR technique, as shown in Figure 3f, g. Compared with the original sample, the voltage ratios of *V_σz_* and *V_σy_* are |*V*_FL‐_ *_σz_*/*V*_DL‐_ *_σy_* | = 0.63 and |*V*_DL‐_ *_σz_*/*V*_FL‐_ *_σy_* | = 0.35, respectively, indicating that the spin current in the *z*‐direction is enhanced under positive gate voltage.

**Figure 4:**
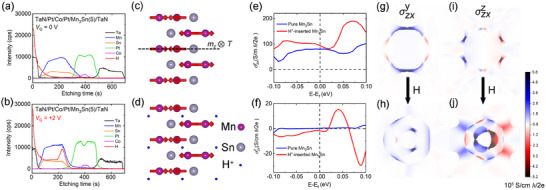
Characterization of the electric field‐induced hydrogen ion migration. The etching time profiles of the SIMS intensity (in counts per second (cps)) of TaN(5)/Pt(1.5)/Co(0.9)/Pt(1)/Mn_3_Sn(5)/TaN(1) under a) *V*_G_ = 0 V and b) *V*_G_ = +2 V. c) Structure of pure Mn_3_Sn, where arrows denote the magnetic moments of Mn and the dashed line is the symmetry plane. d) Structure of H^+^‐inserted Mn_3_Sn. Fermi‐level dependent total e) $\sigma_{zx}^{y}$ and f)$\sigma_{zx}^{z}$ for pure and H^+^‐inserted Mn_3_Sn. Zero energy point corresponds to the neural case. BZ distribution of intrinsic $\sigma_{zx}^{y}$ for g) pure Mn_3_Sn and h) H^+^‐inserted Mn_3_Sn. BZ distribution of $\sigma_{zx}^{z}$ for i) pure Mn_3_Sn and j) H^+^‐inserted Mn_3_Sn. Blue and red colors represent the positive and negative contributions.

The ionic liquid gating effect shows good long‐term stability and reversibility (Figures S21,S22, Supporting Information), which allows us to investigate the mechanism of electrically controlled SOT efficiency by the *ex‐situ* depth‐resolved secondary ion mass spectroscopy (SIMS) as shown in **Figure** **4**a (*V*_G_ = 0 V) and 4b (*V*_G_ = +2 V). For samples under both gate voltages, as the etching time increases, a Ta plateau is first observed, which is followed by a distinct region composed of Mn and Sn elements. The signals of Pt and Co elements are then observed after the Sn plateau, which is consistent with the structure we designed. The intensity of Mn increases slowly with the etching time increases, while the intensity of Sn decreases, which is well in line with the XPS results (Figure 2c). The main difference in element distribution between samples under these two gate voltages is hydrogen. The intensity of the H^+^ signal of the initial sample (*V*_G_ = 0 V) is mainly observed in the TaN layer, which is caused by the absorbed H_2_O.^[^ ^43^ ^]^ However, a clear H^+^ signal exists in the Mn_3_Sn layer and even has a prominent ridge at the Pt/Mn_3_Sn interface for the sample under *V*_G_ = +2 V. It indicates that H^+^ is injected into the Mn_3_Sn layer and accumulated at the Pt/Mn_3_Sn interface with positive gate voltage. As illustrated in Figure 4b, hydrogen ions have fully penetrated the Mn_3_Sn layer, leading us to believe that the electric field effect influences the entire Mn_3_Sn layer. The reason why H^+^ are mainly stored in the Mn_3_Sn layer and cannot be injected into the Pt layer might be that the Pt is too close‐packed and noble to allow the migration or reaction with H^+^.^[^ ^44^ ^]^

Density functional theory is employed to further unveil the microscopic mechanism of the H^+^‐induced enhancement of SOT generation efficiency in Mn_3_Sn. Figure 4c,d show the structures of pure and H^+^‐inserted Mn_3_Sn, where the energetically favored sites for hydrogen impurities are denoted by blue dots. $\sigma_{\alpha \beta }^{\gamma }$ is the calculated spin Hall conductance, where *α*, *β*, and *γ* are directions of the spin‐current propagating, charge current, and spin‐polarization, respectively. Then $\sigma_{zx}^{y}$ and $\sigma_{zx}^{z}$ are utilized to reveal the intensities of *σ_y_* and *σ_z_* spin currents, which are shown in Figure 4e, f, respectively. Compared with the case of pure Mn_3_Sn, both $\sigma_{zx}^{y}$ and $\sigma_{zx}^{z}$ in the H^+^‐inserted system are enhanced around the Fermi energy, which corresponds to a larger SOT efficiency. Figure 4g–j displays Brillouin zone (BZ)‐distributions of $\sigma_{zx}^{y}$ and $\sigma_{zx}^{z}$ in pure and H^+^‐inserted Mn_3_Sn, respectively. The blue and red colors correspond to the positive and negative contributions, and the net spin Hall conductance is a residual effect. Thus, a tiny change in the BZ distribution can lead to significant modulations on the net value. For the pure Mn_3_Sn, the BZ distribution of $\sigma_{zx}^{y}$ exhibits colored circles while that of the H^+^‐inserted system shows colored triangles (Figure 4h), revealing that the insert of H^+^ could induce a symmetry evolution in the system. A similar change is also found in $\sigma_{zx}^{z}$ (Figure 4i,j). A detailed analysis of symmetry evolutions and the computational parameters are provided in Section S18 (Supporting Information). All the results indicate the H^+^‐insertion can enhance the intrinsic spin currents of both in‐plane and out‐of‐plane spin‐polarization, which further increases the SOT efficiency as observed in experiments. Although the theoretical study is based on a crystalline model because of the complexity and immaturity in the calculation for the amorphous system, the H^+^‐insertion‐induced enhancement of spin Hall conductance should be correct qualitatively.

### 2.3 Full‐Electrically Controlled Spin Logic Operations

**Figure 5:**
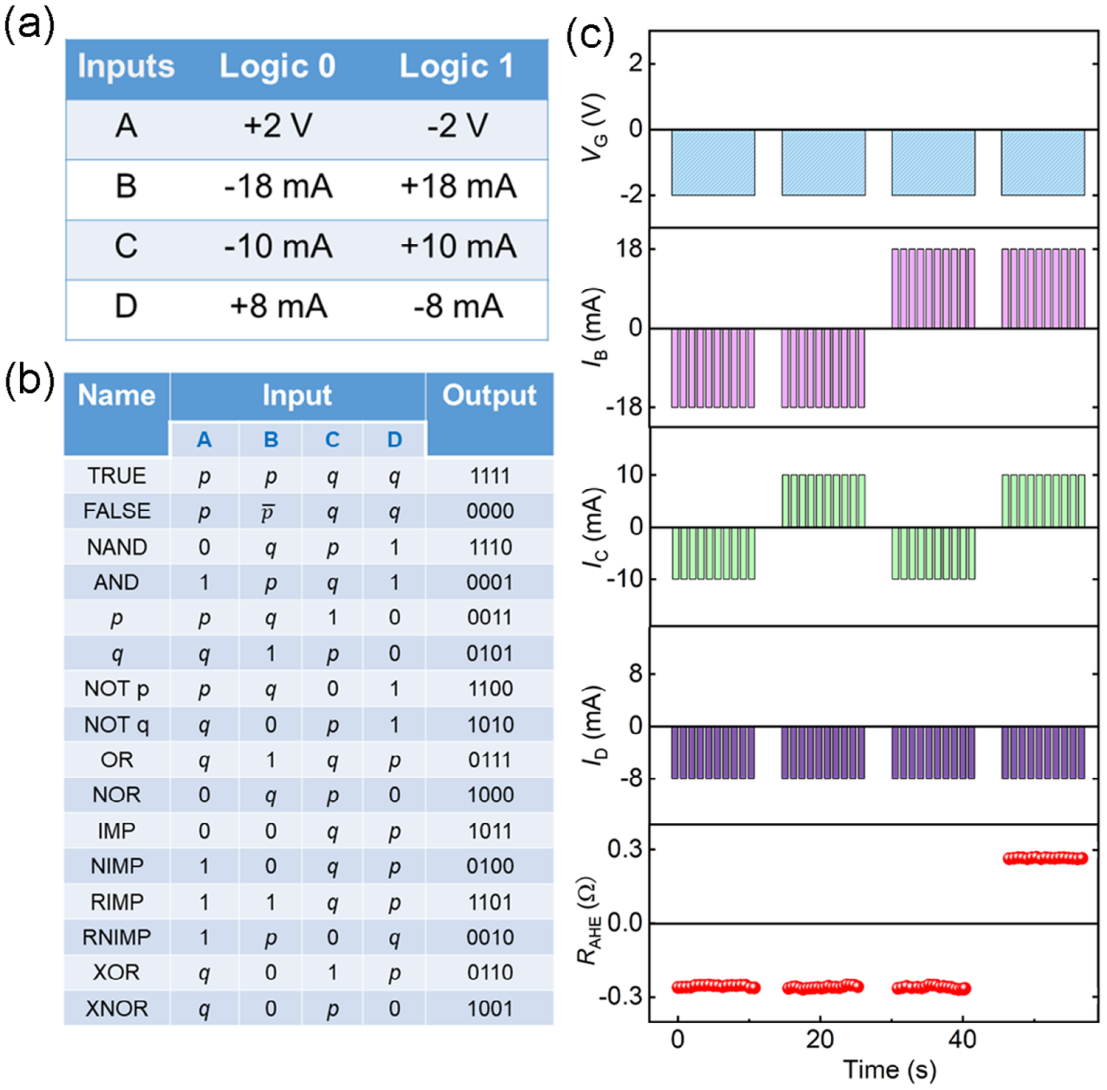
Implementation of Boolean spin logic operations. a) Truth table for the A, B, C, and D input variables. b) Truth table for the four variables to realize 16 Boolean logic operations. c) Experimental results for the “AND” logic function. The variate A represents the *V*_G_. B (*I*_B_), C (*I*_C_), and D (*I*_D_) represent the pulse current (pulse number is 10). When *R*_AHE_ is larger (smaller) than zero, the logic output is “1” (“0”).

Usually, the polarity of SOT‐induced magnetization switching can only be reversed by changing the direction of the external magnetic field once the device is prepared. However, the gate voltage here inverts the switching polarity without any external field, which provides a possibility to achieve logic operations by pure electrical method. The logic operations of *V*_G_ and pulse currents (*I*_B_, *I*_C,_ and *I*_D_) are demonstrated in **Figure** **5**a. Logic inputs *V*_G_, *I*_B_, *I*_C,_ and *I*_D_ are defined as A, B, C, and D, respectively. Each logic input signal has two assignments that represent logic inputs “0” and “1” to realize 16 Boolean logic functions as shown in Figure 5b. For example, we define “*V*_G_ = +2 V” as “A = 0” and “*V*_G_ = −2 V” as “A = 1”. The *R*_AHE_ is used as the logic output “*L*”, which outputs “1” when *R*_AHE_ > 0, while outputs “0” when *R*_AHE_ < 0.

We display the “AND” logic function as a typical example in Figure 5c. The value of “A, B, C, D” are shown in Figure 5a. When logic inputs A and D are both set to be 1, B (*p*) and C (*q*) as input variables implement the “AND” logic function shown as follows. When A = 1, the polarity of magnetization switching is anticlockwise without an external magnetic field. Once B = *p* = 0 (*I*_B_ = −18 mA) is input, the magnetization state is set to the −*z* direction. If D is set to be 1, no matter what the value of C is (*q* = 0 or 1), the final magnetization state stays in the −*z*‐direction, corresponding to the logic output *L* = 0. If B is set to be 1 (*p* = 1, *I*_B_ = +18 mA), the magnetization state tends to the +*z* direction. In the case of C = *q* = 0 (*I*_C_ = −10 mA) and D = 1 (*I*_D_ = −8 mA), the value of *I*_C_ + *I*_D_ is −18 mA, resulting in a magnetization state at −*z* direction (corresponding logic output *L* = 0). In the case of C = *q* = 1 (*I*_C_ = +10 mA) and D = 1 (*I*_D_ = −8 mA), the *I*_C_ + *I*_D_ = 2 mA is too small to change the magnetization state, and the final output is *L* = 1 (e.g., magnetization along +*z* direction). Hence, the logic output *L* = 1 can be obtained only when the *p* and *q* are both set to be 1, which corresponds to the “AND” logic function. More experimental details for logic operations are provided in Figure S23 (Supporting Information), reflecting the stability under varying input conditions and the response time of the logic gates. Similarly, the other 15 Boolean logic operations can also be achieved in Figures S24 and S25 (Supporting Information). The implementation of more complex logic circuits it can be achieved through designing intricate circuit topologies, and multiple logic devices can be combined to realize more sophisticated logic functions.

## 3 Conclusions

In conclusion, we demonstrate that the spontaneous composition gradient in amorphous Mn_3_Sn can generate a DMI field and endow the spin current with a sizeable *σ_z_* component, which allows the SOT‐induced magnetization switching without an external magnetic field. Compared with the previously reported *σ_z_* spin current in crystalline Mn_3_Sn caused by the magnetic spin Hall effect or the interfacial scattering effect, the *σ_z_* spin current in our gradient‐Mn_3_Sn is bulk and robust, which does not call for intricate growth condition but shows a high charge‐to‐spin conversion efficiency. More interestingly, different from the inflexible SOT before, the ionic liquid gating is found to be able to reversibly manipulate the SOT efficiency of the gradient‐Mn_3_Sn layer because of the reversible injection and extraction of hydrogen ions. A positive gate voltage can lead to a ≈10 times larger damping‐like torque efficiency compared with classical heavy metal Pt. Accompanied by the gradient‐Mn_3_Sn SOT efficiency change, the polarity of the field‐free magnetization switching can also be reversed by gate voltage, which is utilized to realize a series of full‐electrically controlled spin logic operations. These results offer an intriguing opportunity to promote the development of SOT‐based in‐memory computing spintronic devices from the perspective of iontronics with low energy dissipation.

## 4 Experimental Section

### Sample Preparation

A series of TaN(5)/Pt(*t*_Pt_)/Co(0.9)/Pt(1)/Mn_3_Sn(*t*_Mn3Sn_)/TaN(1), Ni_81_Fe_19_(15)/Mn_3_Sn(20) and Ni_81_Fe_19_(10)/Pt(10) heterostructures were deposited on thermally oxidized Si substrate by magnetron sputtering at room temperature. The thicknesses of Pt were varied from 1.2 to 3.0 nm, and the thicknesses of Mn_3_Sn were varied from 5 to 40 nm. The base pressure before the deposition was 8.0×10^−5^ Pa. Without special instructions, the Mn_3_Sn layer was deposited by co‐sputtering Mn and Sn using powers of 45 and 5 W, respectively, which produced the Mn_3_Sn layer with a spontaneous composition gradient. The Mn_3_Sn layer without composition gradient was deposited in three steps by co‐sputtering: the power of Sn was fixed at 5 W, while the power of Mn changes as time goes on (43 W for 44 s, then 45 W for 44 s, and finally 47 W for 44 s). The larger gradient‐Mn_3_Sn was deposited in three steps by co‐sputtering: the power of Sn was fixed at 5 W, while the power of Mn changes as time goes on (41 W for 44 s, then 45 W for 44 s and finally 49 W for 44 s).

### Device Fabrication

The samples were patterned into a Hall‐bar shape with an effective area of 20×100 *µ*m by standard photolithography and argon ion etching progress for the transport measurements. Contact electrodes were defined by photolithography and followed by the deposition of Ti(6 nm)/Pt(30 nm). The films were patterned into ST‐FMR devices (Figure S7a, Supporting Information) with device lengths of 80 *µ*m and widths of 20 *µ*m using the same way as Hall‐bar devices. A dope of ionic liquid (N,N‐dienty1‐N‐(2‐methoxyethy1)‐N‐methylammonium bis(triflyoromethylsulfony1)‐imide (DEME‐TFSI)) doped with H_2_O (H_2_O:DEME‐TFSI = 1:50 in volume) was put on the top of device or heterostructure as the electrolyte, through which gate voltage was applied. The gate voltage was applied for 15 min to make the gating effect stable.

### Sample Characterization

The magnetization switching was driven by a series of pulse currents and *R*_AHE_ was characterized by measuring the transverse Hall voltage using a 0.2 mA DC current in the *x* direction. The pulse current width was 50 *µs*. All the measurements were performed at room temperature. The ST‐FMR signals were detected by a lock‐in amplifier. A microwave‐frequency charge current was applied along the longitudinal direction of the device. The frequency and nominal power of the radio frequency current were 4 GHz and 23 dBm, respectively. An in‐plane external magnetic field *H*_ext_ was applied with different angles from the longitudinal direction of the device. In harmonic Hall voltage measurements, the devices were first magnetized to a saturated state by applying a large field (±3.0 T) perpendicular to the film plane. A sinusoidal 0.2 mA AC current of 137 Hz was applied, and the first and second harmonic Hall voltages were simultaneously measured by two lock‐in amplifiers. During the measurement process, an in‐plane field was swept along or vertically to the current direction (*H_x_* or *H_y_*).

Structural properties of the samples were characterized using XRD (Smartlab), with Cu K*α* radiation. The depth‐resolved XPS was done using Al *Kα* (Thermo Scientific K‐Alpha, America). The instrument for SIMS detection was the SIMS 5 time‐of‐flight (TOF) secondary ion mass spectrometer manufactured by IONTOF. Cesium was used to etch the samples gradually, with an energy of 0.5 keV and an etching area of ≈300×300 *µm*. Bismuth ions were employed as the analysis source, with an energy of 30 keV. During the measurements, the sample was gradually etched, and the dynamic changes in the contents of various elements in the films were recorded as a function of the etching time. XMCD in total electron yield mode was performed at Shanghai Synchrotron Radiation Facility (SSRF) beamlines BL08U1A.

### Theoretical Modeling and Numerical Calculations

The density functional theory calculations were conducted with the projector augmented plane‐wave basis (PAW), as implemented in the Vienna ab initio simulation package.^[^ ^45^, ^46^ ^]^ The plane waves were cut off at 550 eV. Exchange and correlations of electrons were approached by the generalized gradient approximations (GGA) with the form suggested by Perdew, Burke, and Ernzerhof.^[^ ^47^ ^]^ The energy convergence criteria for solving the Kohn‐Sham equations is 10^−6^ eV. BZ was sampled with resolutions better than 0.02 Å^−1^, using the scheme of Monkhorst‐Pack, including the Γ‐point.^[^ ^48^ ^]^ The crystal lattice and atomic positions were fully relaxed until the Hellman‐Feynman force was lower than 0.01 eV Å^−1^. Maximally localized wannier functions (MLWF) were computed to extract the effective Hamiltonian as implemented in the WANNIER90 package.^[^ ^49^, ^50^ ^]^

## Conflict of Interest

The authors declare no conflict of interest.

## Author Contributions

B.C. and J.F.H. conceived and supervised the project. M.F.Z. fabricated the samples and performed the measurements. M.F.Z., B.C., T.Y.A., L.L., and J.F.H. analyzed and discussed the experiment results. L.L. performed the theoretical analysis. M.F.Z., B.C., L.L., and J.F.H. wrote the manuscript. All the authors discussed the results and revised the paper.

## Supporting information



Supporting Information

## Data Availability

The data that support the findings of this study are available from the corresponding author upon reasonable request.
