# Supplementary material for: Electrical Manipulation of Field‐Free Magnetization Switching Driven by Spin‐Orbit Torque in Amorphous Gradient‐Mn3Sn
Source: Adv Sci (Weinh). 2025 Mar 24;12(19):2417621. doi: 10.1002/advs.202417621 (PMC12097007; doi:10.1002/advs.202417621)
Supplement: Supplementary file 1 — Supporting Information [file ADVS-12-2417621-s001.docx]

Supporting Information

**Electrical Manipulation of Field-Free Magnetization Switching Driven by Spin-Orbit Torque in Amorphous Gradient-Mn_3_Sn**

*Mingfang Zhang, Bin Cui,** *Taiyu An, Xue Ren, Weikang Liu, Xiangxiang Zhao, Hehe Ding, Zhiyu Zhang, Xu Zhang, Weijie Kuai, Guangjun Zhou, Bin Cheng, Liang Liu,** *and Jifan Hu**

**S1. X-ray diffraction (XRD) results of Mn_3_Sn film**

The XRD data of the TaN(5)/Pt(1.5)/Co(0.9)/Pt(1)/Mn_3_Sn(5)/TaN(1) sample (units in nanometer) is shown in **Figure S1**. Apart from the Si/SiO_2_ substrate peaks, no other diffraction peaks are observed. Given the thicknesses of Pt, Co and Mn_3_Sn layers are very thin, we also measure two thicker samples of Pt(40)/Co(40) and Mn_3_Sn(20). For Pt(40)/Co(40), both diffraction peaks of fcc Pt (111) and hcp Co (002) are found. On the contrary, we find no additional diffraction peaks in Mn_3_Sn(20), which confirms that the Mn_3_Sn thin film prepared at room temperature is amorphous.





**Figure S1.** Structure characterizations of amorphous Mn_3_Sn film. *θ*–2*θ* XRD results of TaN(5)/Pt(1.5)/Co(0.9)/Pt(1)/Mn_3_Sn(5)/TaN(1), Mn_3_Sn(20) and Pt(40)/ Co(40).

**S2. Magnetic properties and anomalous Hall resistance (*R*_AHE_) of Mn_3_Sn film**

**Figure S2a** displays the temperature dependent magnetic susceptibility curve (*χ*–*T*) of Mn_3_Sn(5)/TaN(1) thin film. On heating, a very weak magnetic component vanishes at ~350 K, which is perceived as the Néel temperature for the crystalline non-collinear antiferromagnetic (AFM) Mn_3_Sn. Figure S2b displays the evolution of anomalous Hall effect (AHE) resistance (*R*_AHE_) with sweeping field along *z*-axis (*R*_AHE_–*H*) for Mn_3_Sn(5)/TaN(1) device, where a canted hysteresis loop is observed. Such an AHE could be related to the non-collinear AFM structure which reported in crystalline Mn_3_Sn^[S1,S2]^. The magnitude of *R*_AHE_ change is about one order smaller than that of TaN(5)/Pt(1.5)/Co(0.9)/Pt(1)/Mn_3_Sn(5)/TaN(1), which confirms that the current-induced magnetization switching in this work mainly comes from Co layer with perpendicular magnetic anisotropy (PMA).

**
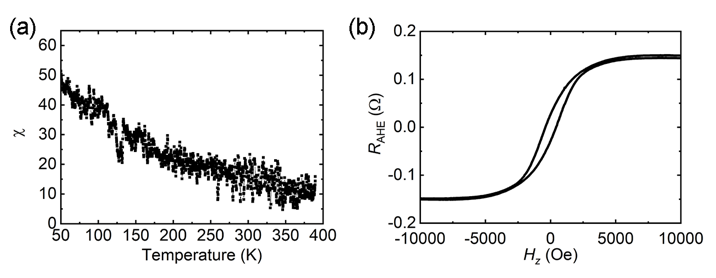
**

**Figure S2.** a) The temperature (K) dependence of magnetic susceptibility curve (*χ*–*T*) of Mn_3_Sn(5)/TaN(1) thin film. b) *R*_AHE_ versus *H_z_* hysteresis loop of Mn_3_Sn(5)/TaN(1) device at 300 K.

**S3.** **Spin-orbit torque (SOT)-induced magnetization switching in Pt/Co/Pt heterostructure**

The SOT-induced magnetization switching measurements are carried out on TaN(5)/Pt(1.5)/Co(0.9)/Pt(1)/TaN(1) and TaN(5)/Pt(3)/Co(0.9)/Pt(1)/TaN(1) without external in-plane magnetic field (Figure S3a and b). There are no obvious magnetization switching in these two samples if no external fields are applied. The results confirm that the Mn_3_Sn layer is crucial for the field-free magnetization switching. The *R*_AHE_ versus pulsed current (*I*) (*R*_AHE_-*I*) loops with the in-plane external magnetic field *H_x_* = +100 Oe are shown in Figure S3c and d. Because of the competition between spin currents from top and bottom Pt layers, the switching polarity is anti-clockwise for the sample with bottom *t*_Pt_ = 1.5 nm, while clockwise once bottom Pt is 3 nm. Thus, the polarity of magnetization switching with positive *H_x_* can be used to reflect the in-plane component of net spin current polarization: clockwise and anti-clockwise polarities mean the in-plane polarization of net spin current is anti-parallel or parallel to that of the spin current generated in the top Pt layer.


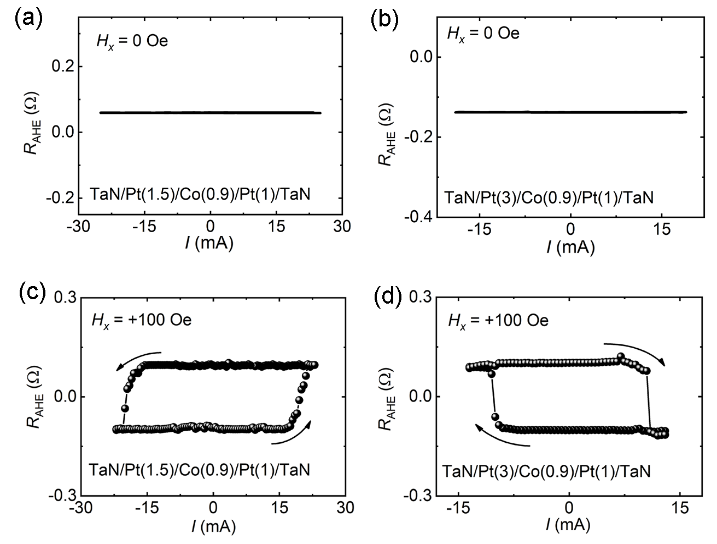


**Figure S3.** SOT-induced magnetization switching of a) TaN(5)/Pt(1.5)/Co(0.9)/Pt(1)/TaN(1) and b) TaN(5)/Pt(3)/Co(0.9)/Pt(1)/TaN(1) without external magnetic field, and c), d**)** with external magnetic field *H_x_* = +100 Oe.

**S4. SOT-induced magnetization switching in TaN(5)/Pt(1)/Co(0.9)/Pt(1)/Mn_3_Sn(5)/ TaN(1)**

The SOT-induced magnetization switching result of TaN(5)/Pt(1)/Co(0.9)/Pt(1)/ Mn_3_Sn(5)/TaN(1) is shown by the *R*_AHE_-*I* loops with *H_x_* = 0 Oe in **Figure S4**. The field-free magnetization switching is observed, demonstrating that the *H*_DMI_ and field-free switching is caused by the Mn_3_Sn, rather than the asymmetry of the Pt layers above and below the Co layer.

**

**

**Figure S4.** The SOT-induced magnetization switching curves with *H_x_* = 0 Oe in TaN(5)/Pt(1)/Co(0.9)/Pt(1)/Mn_3_Sn(5)/TaN(1).

**S5. SOT-induced magnetization switching of heterostructure with different Mn_3_Sn thicknesses**

**Figure S5** shows the *R*_AHE_-*I* loops of TaN(5)/Pt(1.5)/Co(0.9)/Pt(1)/Mn_3_Sn(*t*_Mn3Sn_)/ TaN(1) with different Mn_3_Sn thicknesses, where *t*_Mn3Sn_ is changed from 5 to 40 nm. Field-free magnetization switching is observed in all samples.





**Figure S5.** Field-free SOT-induced magnetization switching in TaN(5)/Pt(1.5)/Co(0.9)/Pt(1)/Mn_3_Sn(*t*_Mn3Sn_)/TaN(1) with different *t*_Mn3Sn_.

**S6. SOT-induced magnetization switching of TaN/Pt(1.5)/Co(0.9)/Pt(1)/Mn_3_Sn(0, 5, 10)/TaN with *H_x_* = +100 Oe**

The *R*_AHE_-*I* loops of TaN/Pt(1.5)/Co(0.9)/Pt(1)/Mn_3_Sn(0, 5, 10)/TaN with *H_x_* = +100 Oe are shown in **Figure S6**. The SOT-induced magnetization switching polarities are anti-clockwise in cases of *t*_Mn3Sn_ = 0 and 5 nm. In these samples, the spin currents are mainly contributed by the top Pt layer (named Pt-dominated). As the *t*_Mn3Sn_ increases to 10 nm, the polarity of SOT-induced magnetization switching turns to be clockwise, suggesting that the spin current is mainly contributed by the Mn_3_Sn and bottom Pt layers (named Mn_3_Sn-dominated). Meanwhile, these results indicate that the polarization (in-plane component) of spin current generated by Mn_3_Sn layer is opposite to that of the Pt layer. On the other hand, if we combine the results in Figure S3 and S6, it can be found that the net in-plane polarizations of spin current are opposite for samples with *t*_Mn3Sn_ = 5 nm and *t*_Mn3Sn_ = 10 nm, but their field-free magnetization switching polarities are the same. It is because their Dzyaloshinskii-Moriya interaction (DMI) field (*H*_DMI_) also have opposite directions (see Figure S14).





**Figure S6.** SOT-induced magnetization switching in TaN/Pt(1.5)/Co(0.9)/Pt(1)/Mn_3_Sn(0, 5, 10)/TaN with *H_x_* = +100 Oe.

**S7. Angle-dependent spin-torque ferromagnetic resonance** (**ST-FMR) measurements**

We carried out angle-dependent ST-FMR measurements of Ni_81_Fe_19_(15)/Mn_3_Sn(20) with different angles *φ* (*φ* is the in-plane angle between magnetic field *H*_ext_ and radio frequency current). The sketch of measurement circuit is shown in **Figure S7**a. The ST-FMR results at several typical angles are shown in Figure S7b-g. The ST-FMR signal *V*_mix._ consists of symmetric (*V*_sym._) and antisymmetric (*V*_anti._) parts. The magnitude of the *V*_sym._ and *V*_anti._ components are extracted from ST-FMR spectrums at different *φ* by using the Equation (S1).^[S3,S4]^

$\text{V}_{\text{mix.}}\text{=}\frac{\text{V}_{\text{sym.}}\text{Δ}\text{H}^{\text{2}}}{\text{[}{\text{(}\text{μ}_{\text{0}}\text{H}_{\text{ext.}}\text{-}\text{μ}_{\text{0}}\text{H}_{\text{FMR}}\text{)}}^{\text{2}}\text{+}\text{Δ}\text{H}^{\text{2}}\text{]}}\text{+}\frac{\text{V}_{\text{anti.}}\text{ΔH}\text{(}\text{μ}_{\text{0}}\text{H}_{\text{ext.}}\text{-}\text{μ}_{\text{0}}\text{H}_{\text{FMR}}\text{)}}{\text{[}{\text{(}\text{μ}_{\text{0}}\text{H}_{\text{ext.}}\text{-}\text{μ}_{\text{0}}\text{H}_{\text{FMR}}\text{)}}^{\text{2}}\text{+}\text{Δ}\text{H}^{\text{2}}\text{]}}$  (S1)

where *μ*_0_, *H*_ext._, *ΔH* and *H*_FMR_ are the permeability of vacuum, the external magnetic field, the spectral width and the ferromagnetic resonance field, respectively.


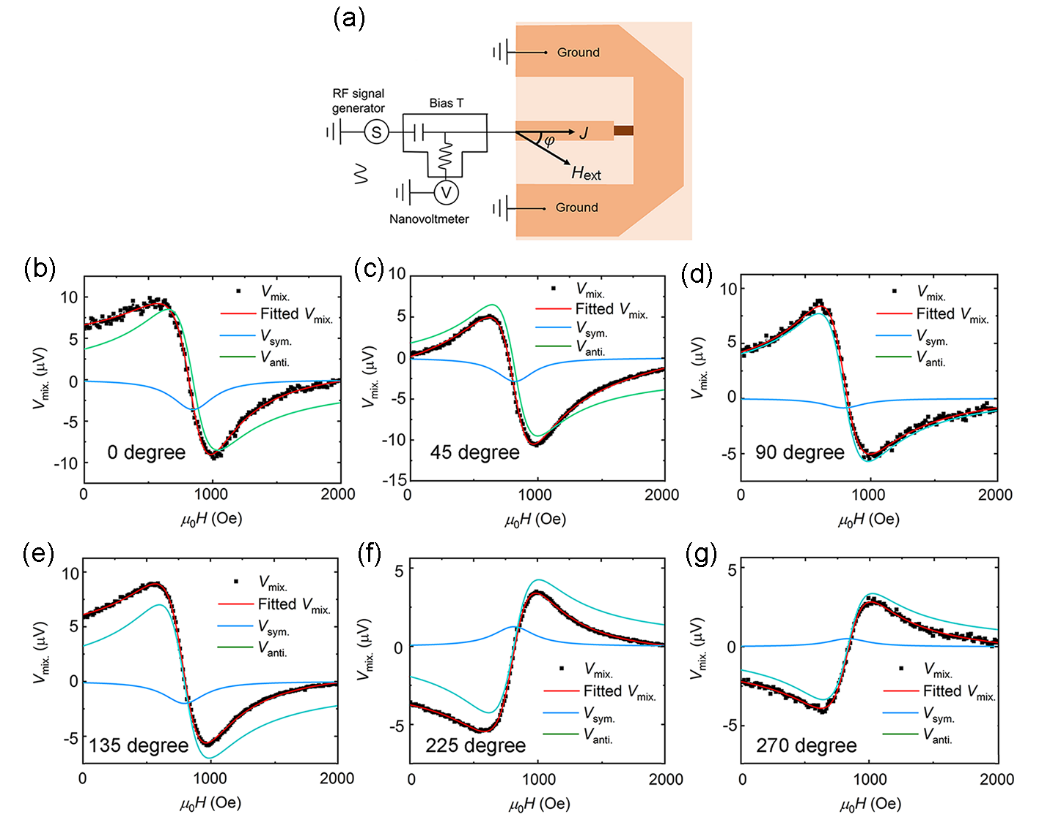


**Figure S7.** ST-FMR measurements in Ni_81_Fe_19_/Mn_3_Sn bilayer. a) Sketch of measurement circuit. Typical ST-FMR results of Ni_81_Fe_19_(15)/Mn_3_Sn(20) at b) *φ* = 0°, c) *φ* = 45°, d) *φ* = 90°, e) *φ* = 135°, f) *φ* = 225° and g) *φ* = 270°. *φ* is the in-plane angle between magnetic field *H*_ext_ and radio frequency current. The frequency of current is 4 GHz.

The angle-dependent ST-FMR measurements are also done in a compared sample of Ni_81_Fe_19_(10)/Pt(10) as shown in Figure S8. The magnitudes of the *V*_sym._ and *V*_anti._ components are extracted from ST-FMR results and summarized as a function of *φ*. The line shapes of *V*_sym._ and *V*_anti._ obey angular dependence (sin2*θ*cos*θ* for *σ_y_* and sin2*θ* for *σ_z_*) and the contribution of in-plane spin polarization (*σ_y_*) and out-of-plane spin polarization (*σ_z_*) could be separated as follows:^[S5,S6]^

$\text{V}_{\text{sym.(}\text{anti.}\text{)}}\text{ }\text{=}\text{ }\text{V}_{\text{σy}}\sin\text{2}\text{φ}\text{∙}\cos\varphi\text{+}\text{V}_{\text{σz}}\sin\text{2}\text{φ}$ (S2)

The voltage ratios of *σ_z_* and *σ_y_*-induced field-like torque and damping-like torque are |*V*_FL-_*_σz_*/*V*_DL-_*_σy_*| = 0.006 and |*V*_DL-_*_σz_*/*V*_FL-_*_σy_*| = 0.009, respectively, which are two orders of magnitude smaller than the values observed in Mn_3_Sn (see Figure 2 of main text).

**
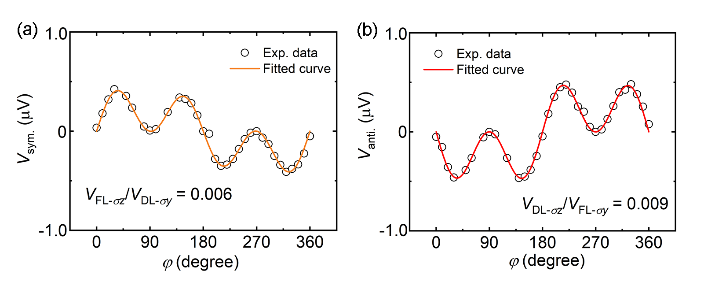
**

**Figure S8.** The angle-dependent ST-FMR signals of Ni_81_Fe_19_(10)/Pt(10) at 4GHz. The magnitude of a) *V*_sym._ and b) *V*_anti._ as a function of the angle *φ* between radio frequency current and magnetic field *H*_ext_. Open circles are the experimental data and the solid lines represent the fitting of data using Equation (S2).

**S8. Properties of** **control sample without Mn-Sn composition gradient**

The control sample of TaN(5)/Pt(1.5)/Co(0.9)/Pt(1)/Mn_3_Sn(5)/TaN(1) without Mn-Sn composition gradient is grown by co-sputtering where the power of Sn is fixed at 5 W and the power of Mn changes as time goes on: 43 W for 44 seconds, then 45 W for 44 seconds and finally 47 W for 44 seconds. According to the depth-resolved X-ray photoelectron spectra (XPS), the atomic ratios of Mn/Sn behave uniform in this control sample, suggesting that the composition gradient is successfully neutralized (**Figure S9**a). Figure S9b shows the *R*_AHE_-*I* loop of this sample. The field-free magnetization switching cannot be realized in this sample any more. And there is a no threshold value of *H*_n_ in Figure S9c, which means that the *H*_DMI_ is negligible in the control sample.


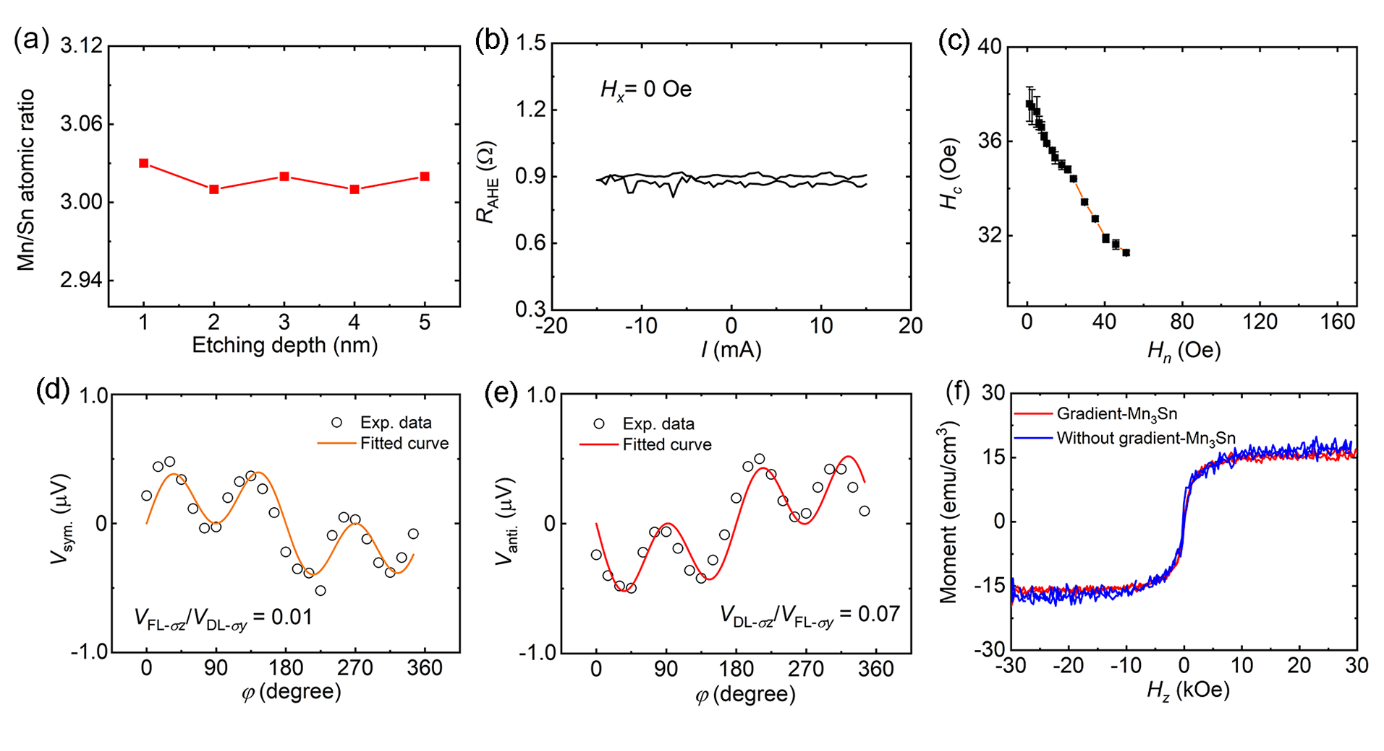


**Figure S9.** Properties of control sample without Mn-Sn composition gradient. a) The dependence of Mn/Sn atomic ratio on the etching depth. The etching depth of 1 nm corresponds to the surface, while that of 5 nm corresponds to the bottom. b) SOT-induced magnetization switching with *H_x_* = 0 Oe. c) The coercive field *H*_c_ as a function of accompanying in-plane field *H*_n_. The sample used for a)–c) is TaN(5)/Pt(1.5)/Co(0.9)/Pt(1)/Mn_3_Sn(5)/TaN(1) without Mn-Sn composition gradient. d) The *V*_sym._ and e) *V*_anti._ as a function of the angle *φ* for Ni_81_Fe_19_/Mn_3_Sn without composition gradient. Open circles are the experimental data and the solid lines represent the fitting of data based on Equation (S2). f) Out-of-plane magnetization versus *H_z_* hysteresis loops of Mn_3_Sn(5)/TaN(1) and with and without composition gradient.

The angular dependence of the *V*_sym._ and *V*_anti._ components of a Ni_81_Fe_19_/Mn_3_Sn without composition gradient are separated and displayed in Figure S9d and e, respectively. The voltage ratios of *σ_z_*- and *σ_y_*-induced field-like torque and damping-like torque are |*V*_FL-_*_σz_*/*V*_DL-_*_σy_*| = 0.01 and |*V*_DL-_*_σz_*/*V*_FL-_*_σy_*| = 0.07, respectively, suggesting that *σ_z_* spin currents are tiny in the sample without composition gradient. The Mn_3_Sn thin films with and without composition gradient show similar *M*-*H* hysteresis loops in Figure S9f, which suggests that the stray field (even exists) should not be the reason for the field-free magnetization switching.

**S9. Properties of control sample** **with opposite Mn-Sn composition gradient**

The control sample with a larger but opposite Mn-Sn composition gradient is grown by co-sputtering, where the power of Sn is fixed at 5 W and the power of Mn changes as time goes on: 41 W for 44 seconds, then 45 W for 44 seconds and finally 49 W for 44 seconds. According to the depth-resolved XPS in **Figure S10**a, the atomic ratios of Mn/Sn gradually decrease from 3.62 at the surface to 2.95 at the bottom. The composition gradient is larger than but opposite to that of the main sample. Figure S10b shows the *R*_AHE_-*I* loop of this sample, where the critical current is 9.5 mA and the polarity of switching is clockwise. The larger composition gradient could reduce the critical current for magnetization switching (11.5 mA for the main sample). And the opposite gradient might be the reason why the polarity of switching is reversed (anti-clockwise for the main sample)


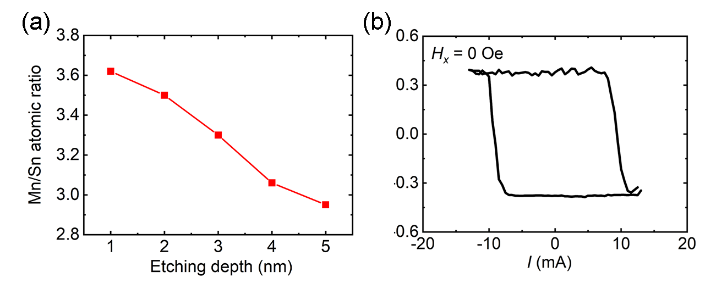


**Figure S10.** Properties of control sample with a larger but opposite Mn-Sn composition gradient. a) The dependence of Mn/Sn atomic ratio on the etching depth for the control sample. The etching depth of 1 nm corresponds to the surface, while that of 5 nm corresponds to the bottom. b) SOT-induced magnetization switching for the control sample with *H_x_* = 0 Oe.

**S10.** **The effect of the interface interactions between gradient-Mn_3_Sn and Pt**

A sample with Cu inserted layer TaN(5)/Pt(1.5)/Co(0.9)/Pt(1)/Cu(1)/Mn_3_Sn(5)/TaN(1) is prepared to investigate the role of Mn_3_Sn/Pt interface in field-free magnetization switching and the formation of gradient-Mn_3_Sn. **Figure S11a** shows its *R*_AHE_-*H_z_* curve and the sample exhibits good PMA. The SOT-induced magnetization switching measurement is then performed with *H_x_* = 0 Oe as shown by *R*_AHE_-*I* loop in Figure S11b. However, the field-free magnetization switching cannot be realized in this sample any more. To figure out the reason for the absence of field-free magnetization switching, the depth-resolved XPS is carried out as shown in Figure S11c. The change of Mn/Sn atomic ratios are much smaller than that of the main sample, suggesting that the Pt layer plays a critical role in formation of gradient-Mn_3_Sn and resulting field-free magnetization switching.


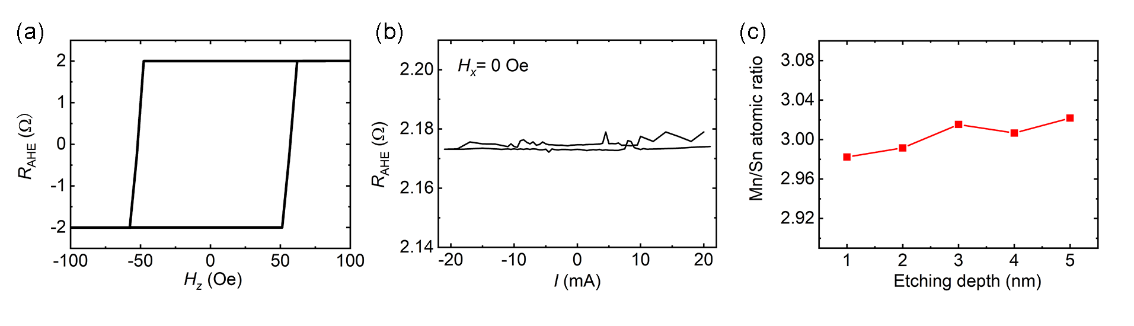


**Figure S11.** a) Dependence of *R*_AHE_ on *H_z_* for TaN(5)/Pt(1.5)/Co(0.9)/Pt(1)/Cu(1)/ Mn_3_Sn(5)/TaN(1). b) SOT-induced magnetization switching with *H_x_* = 0 Oe. c) The dependence of Mn/Sn atomic ratio on the etching depth for the sample. The etching depth of 1 nm corresponds to the surface, while that of 5 nm corresponds to the bottom.

**S11. Characterization of *H*_DMI_**

To quantify the *H*_DMI_ in TaN(5)/Pt(1.5)/Co(0.9)/Pt(1)/Mn_3_Sn(5)/TaN(1), a method based on the magnetic droplet nucleation model was used.^[S7,^^S8]^ The *R*_AHE_ is measured by sweeping the magnetic field at different angles *θ* with respect to the *z*-axis (*R*_AHE_-*H_z_* curves). **Figure S12** illustrates the magnetization switching curves in the negative field range with different angles. If we denote the magnetic field where the magnetization is switched from the “up” state to the “down” state by *H*_sw_, then, the coercive field *H*_c_ and the accompanying in-plane field *H*_n_ can be expressed by *H*_sw_cos*θ* and *H*_sw_sin*θ*, respectively.


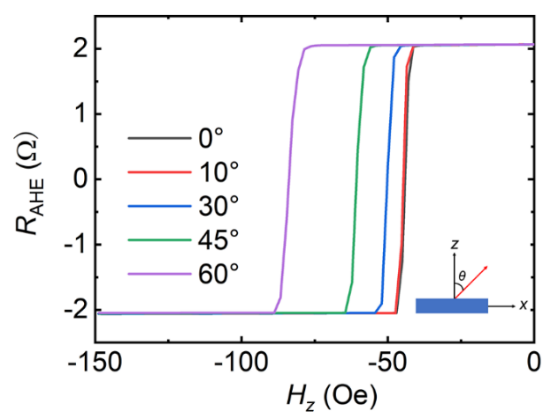


**Figure S12.** *R*_AHE_-*H_z_* curves of TaN(5)/Pt(1.5)/Co(0.9)/Pt(1)/Mn_3_Sn(5)/TaN(1) at different magnetic field angles. The inset shows the definition of *θ* in the *xz* plane.

The *R*_AHE_-*H_z_* hysteresis loops is measured by sweeping the magnetic field at different angles *θ* for TaN(5)/Pt(1.2)/Co(0.9)/Pt(1)/Mn_3_Sn(10)/TaN(1) (**Figure S13**a). The *H*_DMI_ is determined to be around 25 Oe (Figure S13b). The SOT-induced magnetization switching measurement is then performed with different in-plane magnetic fields *H_x_* varying from +100 to –100 Oe as shown by the *R*_AHE_-*I* loops in Figure S13c. The effective in-plane field obtained from magnetization switching measurement is about 30 Oe, which is close to the *H*_DMI_ value.


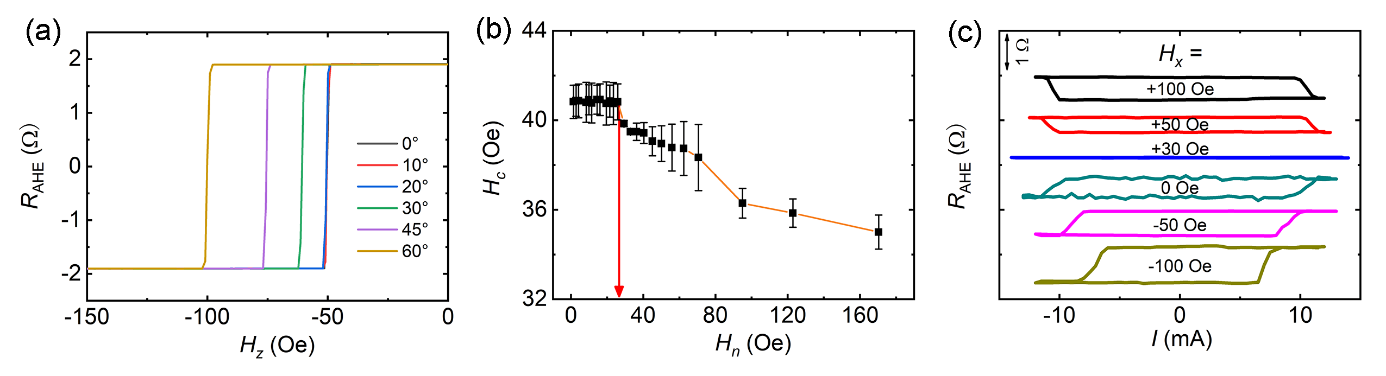


**Figure S13.** *H*_DMI_ and effective in-plane field of TaN(5)/Pt(1.2)/Co(0.9)/Pt(1)/Mn_3_Sn(10)/TaN(1). a) *R*_AHE_-*H_z_* curves at different magnetic field angles *θ*. The angle is same as Figure S12 inset. b) The coercive field *H*_c_ as a function of the accompanying in-plane field *H*_n_. c) The SOT-induced magnetization curves under different *H_x_*.

The directions of *H*_DMI_ for heterostructures with different Mn_3_Sn thicknesses are different. To figure out the direction of *H*_DMI_, we measure the domain wall (DW) velocity as a function of the in-plane magnetic field *H_x_* (*v*-*H_x_* curve).^[S9]^ For TaN(5)/Pt(1.5)/Co(0.9)/Pt(1)/Mn_3_Sn(5)/TaN(1), the DW velocity becomes zero at *H_x_* = –20 Oe (**Figure S14**a). It can be explained by the external magnetic field *H_x_* canceling out the internal DMI effective magnetic field. Differently, the DW velocity becomes zero around *H_x_* = +30 Oe in TaN(5)/Pt(1.2)/Co(0.9)/Pt(1)/Mn_3_Sn(10)/TaN(1) as shown in Figure S14b. Hence, the *H*_DMI_ direction of heterostructure with Mn_3_Sn(10) is opposite to that of heterostructure with Mn_3_Sn(5). This result can explain why these two samples with opposite polarizations of spin current show the same field-free magnetization switching polarity (see Figure S5 and S6). Although the *σ_z_* generated by *H*_DMI_ could well explain all of the experimental results, it should also be noted that the DMI effect itself could break the symmetry and facilitate the field-free switching. The physic image of *σ_z_*, *H*_DMI_, and field-free switching needs to be clarified in the future study.


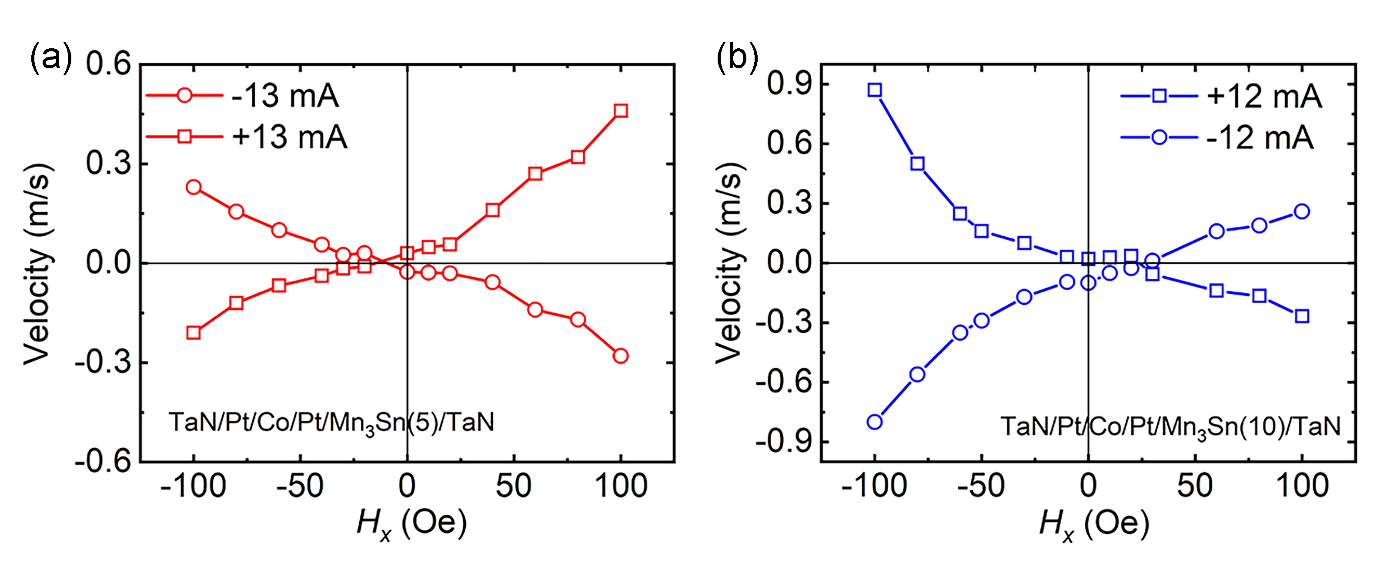


**Figure S14.** Domain wall velocity versus in-plane magnetic field *H_x_* curves of TaN(5)/Pt(1.5)/Co(0.9)/Pt(1)/Mn_3_Sn(*t*_Mn3Sn_)/TaN(1). a) *t*_Mn3Sn_ = 5 nm. b) *t*_Mn3Sn_ = 10 nm. Square and circular symbols correspond to positive and negative pulse currents, respectively.

**S12. X-ray magnetic circular dichroism (XMCD) of** **TaN(5)/Pt(1.5)/Co(0.9)/Pt(1)/ Mn_3_Sn(5)/TaN(1)**

**Figure S15** shows X-ray absorption spectroscopy (XAS) and XMCD for Mn_3_Sn under different magnetic field angles *θ* (*θ* is the angle between magnetic field and *z*-axis direction). XMCD signal is obtained from the difference between the right and left circularly polarized XAS. The clear positive and negative XMCD signals are observed for the Mn *L*_3_ and *L*_2_ edges. Spin and orbital magnetic moments of Mn atoms deduced from sum-rule analyses of XMCD spectra are summarized in Table S1.^[S10,S11]^ With the increase of *θ*, both spin and orbital magnetic moments of Mn are enhanced, indicating that the magnetic moment of Mn has prominent in-plane component.


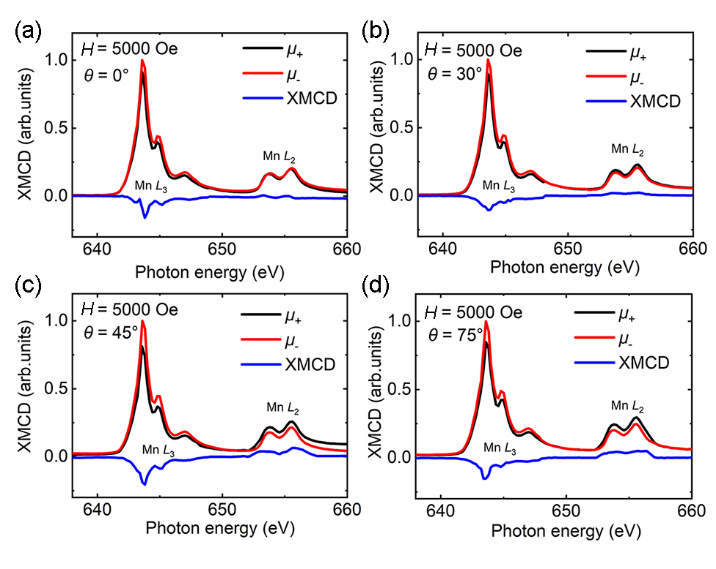


**Figure S15.** XMCD and XAS for TaN(5)/Pt(1.5)/Co(0.9)/Pt(1)/Mn_3_Sn(5)/TaN(1). The XMCD measurements of TaN(5)/Pt(1.5)/Co(0.9)/Pt(1)/Mn_3_Sn(5)/TaN(1) with a magnetic field of 5000 Oe at different angels: a) *θ* = 0°, b) *θ* = 30°, c) *θ* = 45°, d) *θ* = 75°. *θ* is the angle between magnetic field and *z*-axis direction.

**Table S1** Spin and orbital magnetic moments of Mn atoms deduced from sum-rule analyses of XMCD spectra

| *θ* (degree) | 0 | 30 | 45 | 75 |
| --- | --- | --- | --- | --- |
| *m*_spin_ (*μ*B) | 0.19 | 0.27 | 0.39 | 0.47 |
| *m*_orb_ (*μ*B) | 0.16 | –1.08 | –1.37 | –1.88 |

**S13. Ionic liquid gating effect on SOT-induced magnetization switching in TaN(5)/Pt(1.5)/Co(0.9)/Pt(1)/Mn_3_Sn(5)/TaN(1) with external magnetic field**

The *R*_AHE_-*I* loops of TaN(5)/Pt(1.5)/Co(0.9)/Pt(1)/Mn_3_Sn(5)/TaN(1) under gate voltages (*V*_G_) of 0 and +2 V with *H_x_* = +100 Oe are shown in **Figure S16**. The polarity of SOT-induced magnetization switching is anti-clockwise when *V*_G_ is 0 V, while the switching polarity is revered to clockwise under *V*_G_ = +2 V. Combined with the results in Figure S6, we know that the in-plane component of spin current polarization is dominated by Pt at *V*_G_ = 0 V, but dominated by Mn_3_Sn at *V*_G_ = +2 V.

**
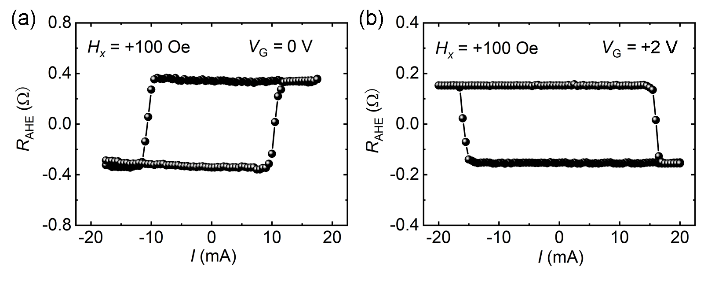
**

**Figure 16.** SOT-induced magnetization switching with external field. *R*_AHE_-*I* loops of TaN(5)/Pt(1.5)/Co(0.9)/Pt(1)/Mn_3_Sn(5)/TaN(1) with *H_x_* = +100 Oe at a) *V*_G_ = 0, b) +2 V.

**S14.** **Measurement and calculation of SOT efficiency for TaN(5)/Pt(1.5)/Co(0.9)/Pt(1)/ Mn_3_Sn(5)/TaN(1)**

The harmonic voltage measurements with parallel or perpendicular magnetic fields (*H_x_* or *H_y_*) are carried out on TaN(5)/Pt(1.5)/Co(0.9)/Pt(1)/Mn_3_Sn(5)/TaN(1) under different *V*_G_. The in-phase first (*V*_ω_) and the out-of-phase second harmonic voltage (*V*_2ω_) are recorded by lock-in amplifiers during the sweeping of the magnetic field *H_x_* and *H_y_* as shown in **Figure S17**a–j. The damping-like field (*H*_DL_) and field-like field (*H*_FL_) are calculated as follows:^[S12-S14]^

$\text{H}_{\text{FL}}\text{ ≈ }\frac{\sin\text{2}\text{θ}_{\text{H}}}{\text{2}\text{ξ}\text{H}_{\text{K}}}\text{[1/(}\frac{\text{∂}\text{V}_{\text{ω}}}{\text{∂}\text{H}}\text{)(}\frac{\text{∂(1/}\text{V}_{\text{2ω}}\text{)}}{\text{∂}\text{H}}\text{)]}$ (S3)

$\text{H}_{\text{DL}}\text{ = –2(}\frac{\text{∂}\text{V}_{\text{2ω}}^{\text{ xx}}}{\text{∂}\text{H}}\text{/}\frac{\text{∂}^{\text{2}}\text{V}_{\text{ω}}^{\text{ xx}}}{\text{∂}\text{H}^{\text{2}}}\text{)}$ (S4)

where $\text{H}_{\text{K}}\text{ ≡}\text{ }{\text{2}\text{K}}_{\text{Eff}}/\text{M}_{\text{s}}$ is the out-of-plane anisotropy field, $\text{K}_{\text{Eff}}$ is the effective out-of-plane anisotropy energy, and *ξ* is Δ*R*_PHE_/Δ*R*_AHE_. Δ*R*_PHE_ and Δ*R*_AHE_ are changes of planar Hall effect (PHE) resistance (Figure S17k) and AHE resistance, respectively. The value of *ξ* is not changed by *V*_G_ (*ξ* = 0.1).

Based on the results of the harmonic voltage measurements, we obtain the values of SOT effective fields *H*_DL(FL)_ in TaN(5)/Pt(1.5)/Co(0.9)/Pt(1)/Mn_3_Sn(5)/TaN(1) (main sample) under different *V*_G_. Through the harmonic voltage measurements in the same current, we also get the values of *H*_DL(FL)_ in a control sample of TaN(5)/Pt(1.5)/Co(0.9)/Pt(1)/TaN(1). Then the Mn_3_Sn layer and the Pt(1.5)/Co(0.9)/Pt(1) layers are regarded as parallel circuits in shunting model (the TaN layer is treated as insulating), which means both SOT effective fields *H*_DL(FL)_ and current are consisted by Pt/Co/Pt part ($\text{H}_{\text{Pt/Co/Pt}}^{\text{DL(FL)}}$ and *I*_Pt/Co/Pt_) and Mn_3_Sn part ($\text{H}_{\text{Mn3Sn}}^{\text{DL(FL)}}$ and *I*_Mn3Sn_) in the main sample. The resistivities of materials: *ρ*_Mn3Sn_ = 347.5 *μ*Ω∙cm, *ρ*_Pt_ = 33.6 *μ*Ω∙cm, *ρ*_Co_ = 91.1 *μ*Ω∙cm are used for the calculation of *I*_Pt/Co/Pt_ and *I*_Mn3Sn_. The *H*_DL(FL)_ values obtained from the control sample are scaled according to the current in Pt/Co/Pt part of main sample to get the values of $\text{H}_{\text{Pt/Co/Pt}}^{\text{DL(FL)}}$. We assume $\text{H}_{\text{Mn3Sn}}^{\text{DL(FL)}}$ to be positive and then $\text{H}_{\text{Pt/Co/Pt}}^{\text{DL(FL)}}$ is set to be negative due to their opposite SOT-induced magnetization switching polarities. The SOT effective fields of $\text{H}_{\text{Total}}^{\text{DL}\text{(FL)}}$, $\text{H}_{\text{Pt/Co/Pt}}^{\text{DL(FL)}}$, and $\text{H}_{\text{Mn3Sn}}^{\text{DL(FL)}}$ are summarized in Table S2. Here we do not consider the possible but tiny change of $\text{H}_{\text{Pt/Co/Pt}}^{\text{DL(FL)}}$ with gate voltage due to the following reasons: 1) Pt is cover by 5 nm-thick Mn_3_Sn, which absorbs overwhelming H^+^ at positive gate voltage (see secondary ion mass spectroscopy results in the main text); 2) the ionic liquid gating effect on Pt is dramatically weakened once *t*_Pt_ is thicker than 1 nm.^[S15]^ These values are then used for the calculation of SOT efficiency *β = H*_DL(FL)_/*J* in Figure 3d and e of the main text, where *J* is the current density for the whole heterostructure or Mn_3_Sn layer.


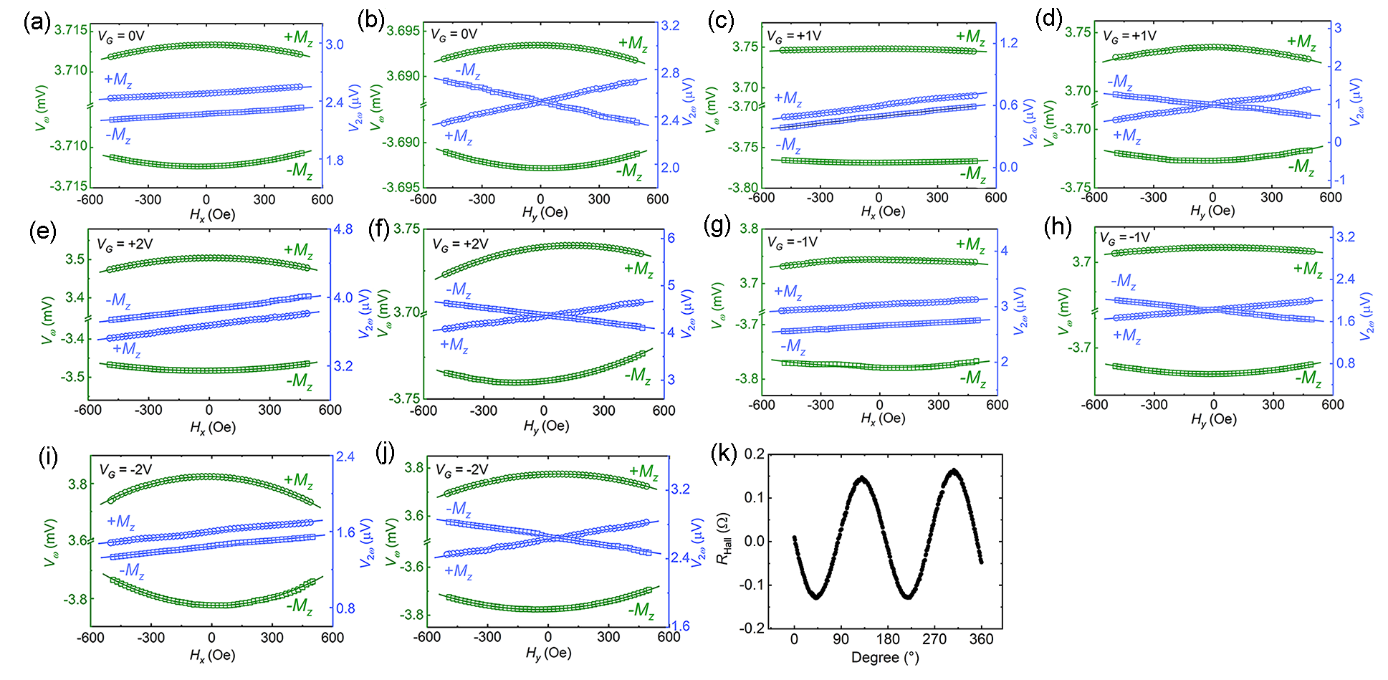


**Figure S17.** Harmonic voltage measurements of TaN(5)/Pt(1.5)/Co(0.9)/Pt(1)/ Mn_3_Sn(5)/TaN(1). Field dependence of first-(green colors, left axis) and second-(blue colors, right axis) harmonic signals on the magnetic field *H_x_* ((a), (c), (e), (g) and (i)) and *H_y_* ((b), (d), (f), (h) and (j)): a), b) *V*_G_ = 0, c), d) *V*_G_ = +1 V, e), f) *V*_G_ = +2 V, g), h) *V*_G_ = −1 V, i), j) *V*_G_ = −2 V. The open circles and square correspond to out-of-plane magnetization components of +*M_z_* and –*M_z_*, respectively. Open symbols are the experimental data and the solid lines represent the fitting of the data based on Equation (S3) and (S4). The input current *I*_ac_ is 0.2 mA. *V*_G_ is applied with 15 minutes to be stable. k**)** In-plane angle-dependent transverse resistance (PHE resistance) at 3 T.

**Table S2** The values of the *H*_DL(FL)_ under different *V*_G_ in whole heterostructure, Pt(1.5)/Co(0.9)/Pt(1) layer, Mn_3_Sn layer

| *V*_G_ | $\text{H}_{\text{Total}}^{\text{DL}}$(Oe) | $\text{H}_{\text{Total}}^{\text{FL}}$(Oe) | $\text{H}_{\text{Pt/Co/Pt}}^{\text{DL}}$(Oe) | $\text{H}_{\text{Pt/Co/Pt}}^{\text{FL}}$(Oe) | | $\text{H}_{\text{Mn3Sn}}^{\text{DL}}$(Oe) | $\text{H}_{\text{Mn3Sn}}^{\text{FL}}$(Oe) |
| --- | --- | --- | --- | --- | --- | --- | --- |
| 0 V | –0.45 | 0.15 | –0.67 | | –0.27 | 0.21 | 0.12 |
| +1 V | –0.25 | 0.06 | –0.67 | | –0.27 | 0.41 | 0.21 |
| +2 V | 0.06 | 0.02 | –0.67 | | –0.27 | 0.7 | 0.28 |
| –1 V | 0.10 | 0.04 | –0.67 | | –0.27 | 0.77 | 0.31 |
| –2 V | –0.52 | 0.12 | –0.67 | | –0.27 | 0.14 | 0.15 |

**S15. Electrical control of SOT in heterostructure with 10 nm-thick Mn_3_Sn**


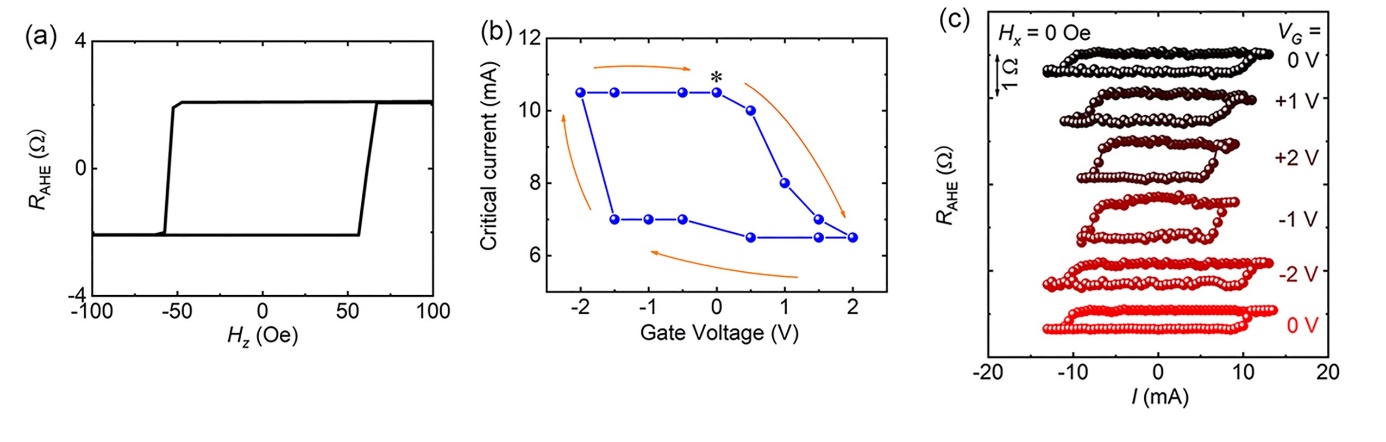


**Figure S18.** Electrical manipulation of SOT-induced magnetization switching in TaN(5)/Pt(1.2)/Co(0.9)/Pt(1)/Mn_3_Sn(10)/TaN(1). a) Dependence of *R*_AHE_ on *H_z_*. b) The evolution of the critical current under different *V*_G_. The“*” represents the initial state before applying the *V*_G_. c) *R*_AHE_-*I* loops for the device under different *V*_G_ without an external magnetic field. *V*_G_ is applied in the sequence of 0 V → +2 V → 0 V → –2 V → 0 V.

**Figure S18**a shows the *R*_AHE_ of TaN(5)/Pt(1.2)/Co(0.9)/Pt(1)/Mn_3_Sn(10)/TaN(1) and the sample exhibits good PMA. Figure S18c shows *R*_AHE_-*I* loops under different *V*_G_ without external in-plane magnetic field. The SOT-induced field-free magnetization switching is achieved at different *V*_G_ with the same switching polarity, while the magnitudes of critical current differ. As summarized in Figure S18b, the critical current decreases from 10.5 to 6.5 mA when *V*_G_ increases from 0 to +2 V. On the contrary, the application of negative *V*_G_ induces a remarkable increase in critical current (*I* = 10.5 mA at *V*_G_ = −2 V), suggesting that the IL gating effect is almost reversible.

The harmonic voltage measurements with *H_x_* and *H_y_* under different *V*_G_ are studied to investigate the electrical manipulation of SOT efficiency in TaN(5)/Pt(1.2)/Co(0.9)/Pt(1)/Mn_3_Sn(10)/TaN(1). **Figure S19** shows the curves of the harmonic voltages for samples under *V*_G_ = 0, ±1, ±2, and −2.5 V. The *H*_DL_ and *H*_FL_ can be calculated by Equations S3 and S4. Meanwhile the SOT efficiencies of the whole heterostructure and Mn_3_Sn layer are calculate base on a shunting model above and shown in **Figure S20**. The $\text{β}_{\text{Mn3Sn}}^{\text{DL}}$ = 85.2 Oe per 10^7^A/cm^2^ and $\text{β}_{\text{Mn3Sn}}^{\text{FL}}$ = 70.7 Oe per 10^7^A/cm^2^ are obtained at *V*_G_ = 0 V, which increase to $\text{β}_{\text{Mn3Sn}}^{\text{DL}}$ =152.1 Oe per 10^7^A/cm^2^ and $\text{β}_{\text{Mn3Sn}}^{\text{FL}}$ = 137.3 Oe per 10^7^A/cm^2^ with the application of *V*_G_ = +2 V. Then the $\text{β}_{\text{Mn3Sn}}^{\text{DL}}$ and $\text{β}_{\text{Mn3Sn}}^{\text{FL}}$ are decreased to 78.6 and 72.7 Oe per 10^7^A/cm^2^ when *V*_G_ is −2.5 V, which are close to the initial ones. Similarly, positive and negative gate voltages increase and decrease the SOT efficiencies of the whole heterostructure ($\text{β}_{\text{Total}}^{\text{DL}}$ and $\text{β}_{\text{Total}}^{\text{FL}}$), respectively, because the SOT of heterostructure is dominated by the 10-nm thick Mn_3_Sn layer. These results reconfirm the gating effect on the SOT efficiency of Mn_3_Sn.


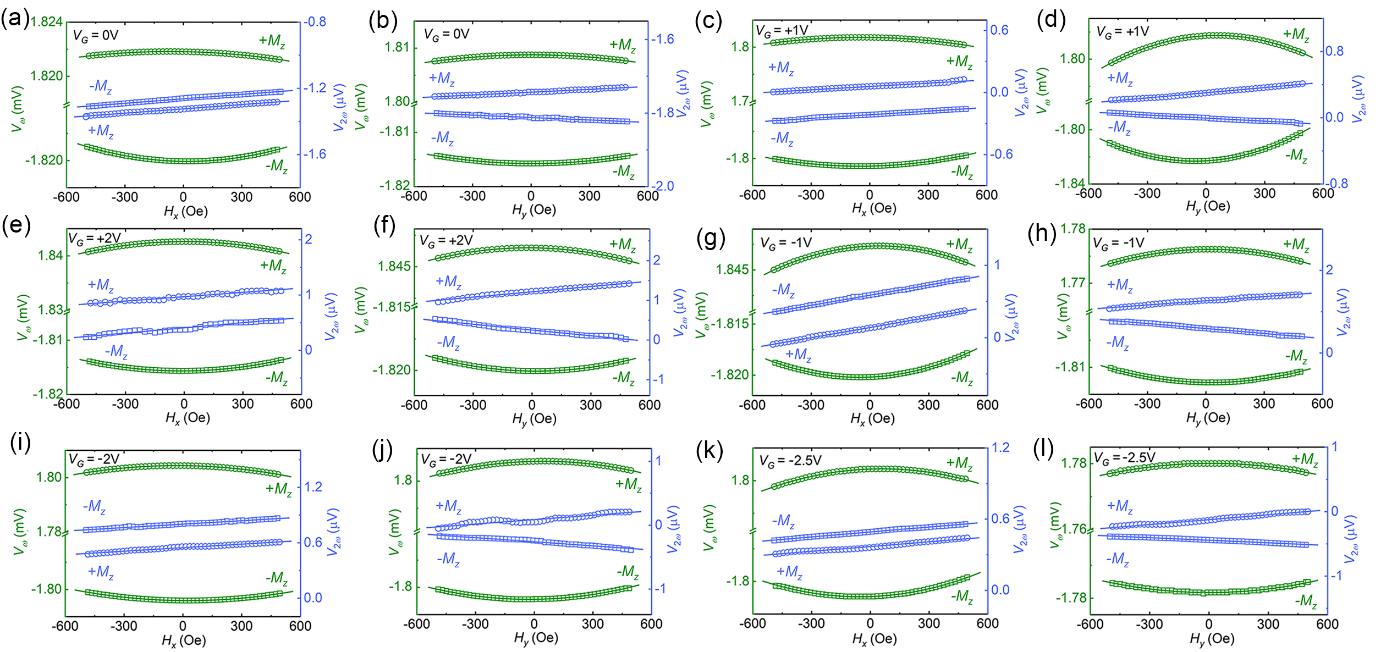


**Figure S19.** Harmonic voltage measurements of TaN(5)/Pt(1.2)/Co(0.9)/Pt(1)/ Mn_3_Sn(10)/TaN(1). Field dependence of first-(green colors, left axis) and second-(blue colors, right axis) harmonic signals on the magnetic field *H_x_* ((a), (c), (e), (g), (i) and (k)) and *H_y_* ((b), (d), (f), (h), (j) and (l)): a), b) *V*_G_ = 0, c), d) *V*_G_ = +1 V, e), f) *V*_G_ = +2 V, g), h) *V*_G_ = −1 V, i), j) *V*_G_ = −2 V, k), l) *V*_G_ = −2.5 V. The open circles and square correspond to out-of-plane magnetization components of +*M_z_* and –*M_z_*, respectively. Open symbols are the experimental data and the solid lines represent the fitting of the data based on Equation (S3) and (S4). The input current *I*_ac_ is 0.2 mA.


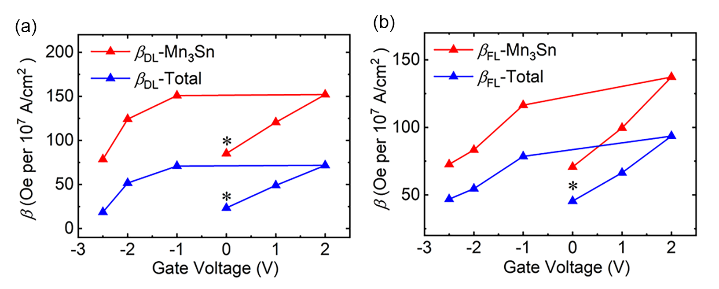


**Figure S20.** The SOT efficiencies of Mn_3_Sn and whole heterostructure under different *V*_G_: a) *β*_DL_ and b) *β*_FL_.

**S16. The long-term stability for the modulating of SOT efficiency**

To verify the long-term stability of gate voltage effect, the variation of the critical current for magnetization switching with the application time of *V*_G_ is studied. **Figure S21** shows the critical current changes with the applying time of *V*_G_, where a constant *V*_G_ (*V*_G_ = +2 V) is applied for 20 minutes and then removed. The critical current decreases when the *V*_G_ is applied, and then reaches a nearly constant value after the *V*_G_ is applied for 15 minutes. Subsequently, the gating effect on the critical current maintains even after the *V*_G_ is removed for 3 days, which exhibits good stability and non-volatile character.


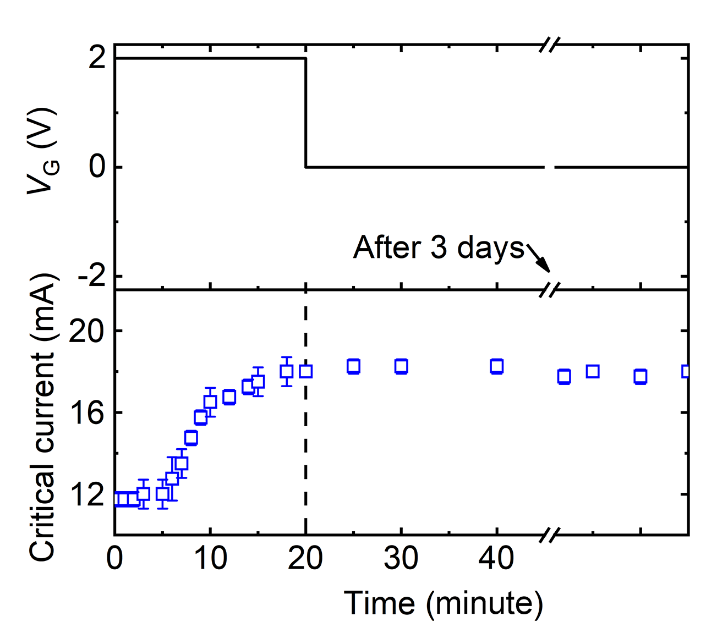


**Figure S21.** The critical current for magnetization switching as a function of *V*_G_ application time (bottom panel). The top panel is a schematic of the applied *V*_G_ versus time, where *V*_G_ = +2 V is applied for 20 minutes and the removed.

**S17. The reversibility for the modulating of SOT efficiency**

**Figure S22** shows the *R*_AHE_-*I* loops under different *V*_G_ with *H_x_* = 0 Oe. The *V*_G_ is applied in the sequence of 0 V → +1.5 V → –1.5 V → +2.5 V → −2.5 V. The dependence of critical current on the *V*_G_ is summarized in Figure S22b. The virgin device (*V*_G_ = 0 V) exhibits a critical current of 11.5 mA. The value of critical current increases to 15 mA when the device is processed by *V*_G_ = +1.5 V. Afterwards, with the application of *V*_G_ = –1.5 V, the critical current decreases to 12.0 mA, which is closed to the virgin state. Successively, the critical current dramatically increases to 18.0 mA under *V*_G_ = +2.5 V accompanied by the reversal of switching polarity (clockwise). While the application of *V*_G_ = −2.5 V induces an anti-clockwise magnetization switching with a remarkable decrease in the critical current to 11.6 mA, confirming that the gating effect is almost reversible.


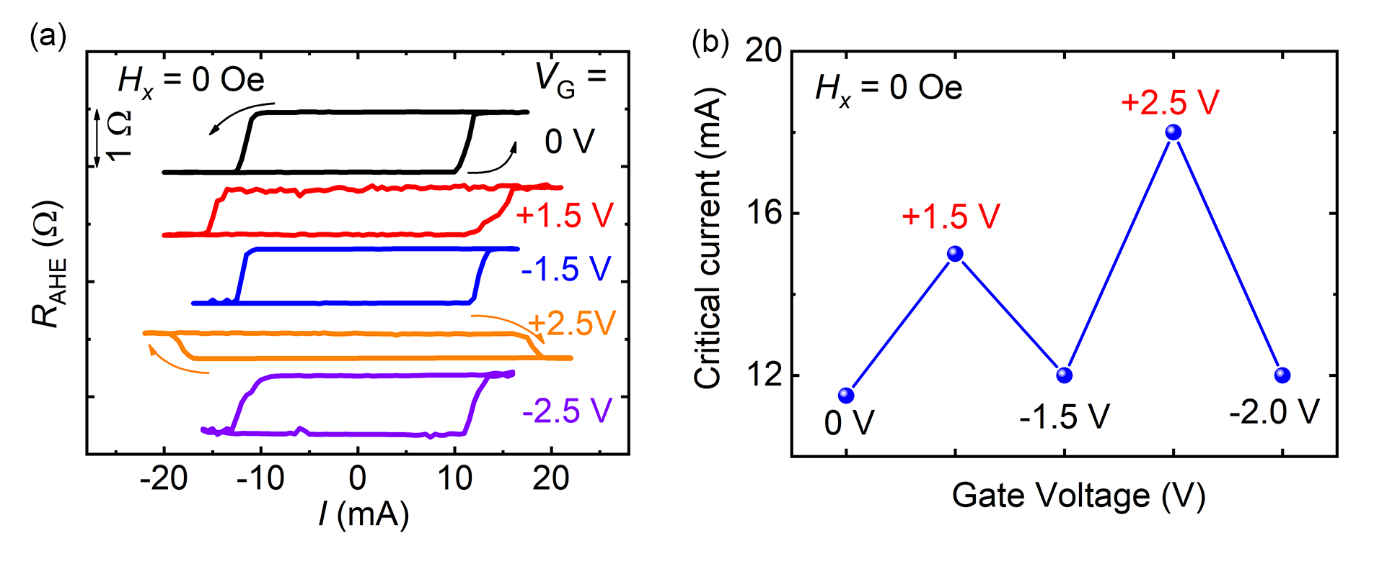


**Figure S22.** Modulations on the current-induced magnetization switching through gate voltage. a) *R*_AHE_ versus *I* loops for TaN(5)/Pt(1.5)/Co(0.9)/Pt(1)/Mn_3_Sn(5)/TaN(1) under different *V*_G_ with *H_x_* = 0 Oe. The gate voltage is applied in the sequence of 0 V → +1.5 V → –1.5 V → +2.5V → –2.5 V. b) The evolution of the critical current with *V*_G_.

**S18.** **Density functional theory of the H^+^-insertion induced intrinsic** $\text{σ}_{\text{zx}}^{\text{z}}$ **by symmetry evolution**

Up to the linear responses, the generated spin currents are related to the electric field by:

$\text{J}_{\text{S}\text{,}\text{α}}^{\text{γ}}\text{ }\text{=}{\text{ }\text{σ}}_{\text{αβ}}^{\text{γ}}\text{E}_{\text{β}}$ (S5)

where $\text{J}_{\text{S}\text{,}\text{α}}^{\text{γ}}$ is the spin-current propagating in *α*-direction with the spin-polarization along *γ*-direction. $\text{E}_{\text{β}}$ is the dc-electric field in *β*-direction. $\text{σ}_{\text{αβ}}^{\text{γ}}$ is the spin Hall conductance, whose intrinsic part can be estimated via Kubo formalism:

$\text{σ}_{\text{αβ}}^{\text{γ}}\text{ }\text{=}\text{ }\frac{\text{2}\text{e}}{\text{ℏ}}\text{ }\text{Im}\sum_{\text{mn}} \int_{\text{BZ}} \frac{\text{dk}}{\left( \text{2}\text{π} \right)^{\text{2}}}\text{ }\text{f}_{\text{n}}\frac{\text{j}_{\text{α}\text{,}\text{nm}}^{\text{γ}}\text{v}_{\text{β}\text{,}\text{mn}}}{\left( \text{E}_{\text{m}}\text{-}\text{E}_{\text{n}}\text{+}\text{iℏ}\text{/}\text{τ} \right)^{\text{2}}}$ (S6)

here, *m, n* label band, $\text{v}_{\text{α}\text{,}\text{mn}}$ is the matrix element of velocity operator. $\text{j}_{\text{α}}^{\text{γ}}\text{ }\text{=}\text{ }\left\{ \text{s}^{\text{γ}}\text{,}\text{v}_{\text{α}} \right\}\text{ }\text{=}\text{ }\left( \text{v}_{\text{α}}\text{s}^{\text{γ}}\text{ }\text{+}\text{ }\text{s}^{\text{γ}}\text{v}_{\text{α}} \right)\text{/2}$ is the spin-current operator. $\text{E}_{\text{m}}$ is the band energy. The explicit *k*-dependents are omitted for concise.

The H^+^-insertion induced intrinsic $\text{σ}_{\text{zx}}^{\text{z}}$ is attributed to the symmetry evolution. As clearly indicated by the geometry of pristine Mn_3_Sn, a composite symmetry $\text{m}_{\text{z}}\text{⊗}\text{T}$ is preserved, where *m_z_* is the mirror reflection and *T* is time reversal. Under *m_z_* transformation, the quantity *s_z_*, *v_x_* and *v_z_* are even, even and odd, respectively. Under time reversal, these terms become complex conjugate with extra negative sign. So, the nominator of Equation (S6) $\text{N}_{\text{αβ}}^{\text{γ}}\text{ }\text{=}{\text{ }\text{j}}_{\text{α}}^{\text{γ}}\text{v}_{\text{β}}$ transforms as $\text{m}_{\text{z}}\text{⊗}\text{T}\text{ }\text{N}_{\text{αβ}}^{\text{γ}}\left( \text{k}_{\text{x}}\text{,}{\text{ }\text{k}}_{\text{y}}\text{,}{\text{ }\text{k}}_{\text{z}} \right)$ = $\text{N}_{\text{αβ}}^{\text{γ}\text{,*}}\left( \text{–}\text{k}_{\text{x}}\text{,}\text{ }\text{–}\text{k}_{\text{y}}\text{,}{\text{ }\text{k}}_{\text{z}} \right)$. The imaginary part of nominator is odd under $\text{m}_{\text{z}}\text{⊗}\text{T}$ symmetry. The denominators in Equation (6) is unchanged under $\text{m}_{\text{z}}\text{⊗}\text{T}$. After integrations on Brillouin zone (BZ), $\text{σ}_{\text{zx}}^{\text{z}}$ vanishes. On the other hand, inserted H^+^ can break the $\text{m}_{\text{z}}\text{⊗}\text{T}$ symmetry, removing the constrains on $\text{σ}_{\text{zx}}^{\text{z}}$. These results indicate that the H^+^-insertion induced symmetry-breaking can produce intrinsic *σ_z_* spin currents in Mn_3_Sn.

For the DFT calculations on the ground state charge density and one-particle wavefunctions, the energy convergence criteria for solving the self-consistent Kohn-Sham equation is 10^−6^ eV, and Brillouin zone (BZ) is sampled with resolutions better than 0.02 Å^−1^ for both systems with and without hydrogen insertions. Then the Hamiltonians in the local basis of maximally localized Wannier orbitials are constructed, and the spin Hall conductance is computed via Kubo’s formula based on the Wannier Hamiltonians. The *k*-point sampling for the BZ integration of Kubo’s formula is 140×140×160. The difference between the computed results with smaller *k*-point sampling 70×70×80 and the presented ones are less than 5%, indicating that the utilized *k*-point sampling is sufficient to reach the numerical convergence.

**S19. Boolean spin logic operations.**

**Figure S23** shows the experimental data of the implementation of an “AND” logic function. Inputs B and C are logic inputs, while inputs A and D here define the “AND” function. When the final *R*_AHE_ > 0, the logic output is set as “1”, while logic output is set to be “0” once *R*_AHE_ < 0. Firstly, input A is fixed to be 1, e.g. *V*_G_ = –2 V, which determines the current-induced magnetization switching polarity to be anticlockwise. Subsequently, the input B = 0 (*I*_B_ = –18 mA) drives the magnetization down with a negative *R*_AHE_ (Figure S23a and b), while the input B = 1 (*I*_B_ = +18 mA) drives the magnetization up with a positive *R*_AHE_ (Figure S23c and d). Finally, a combined inputs C and D (*I*_C_ + *I*_D_) determines the logic output, where *I*_C_ = +10 or –10 mA (input C = 1 or 0) and *I*_D_ is fixed to be –8 mA. *I*_C_ + *I*_D_ = –18 mA stabilizes the magnetization in –*z* direction with *R*_AHE_ < 0 and logic output is “0” (Figure S23a and c). While *I*_C_ + *I*_D_ = +2 mA is too small to change the magnetization state, the magnetization is stable in –*z* in Figure S23b (*R*_AHE_ < 0, logic output is “1”) and +*z* in Figure S23d (*R*_AHE_ > 0, logic output is “1”). Hence, the logic output “1” can be obtained only when inputs B and C are both set to be 1 (Figure S23d), which corresponds to the “AND” logic function. Under different input conditions, the *R*_AHE_ consistently exhibits stable switching, and the response time of the logic gate is approximately 1 minute.


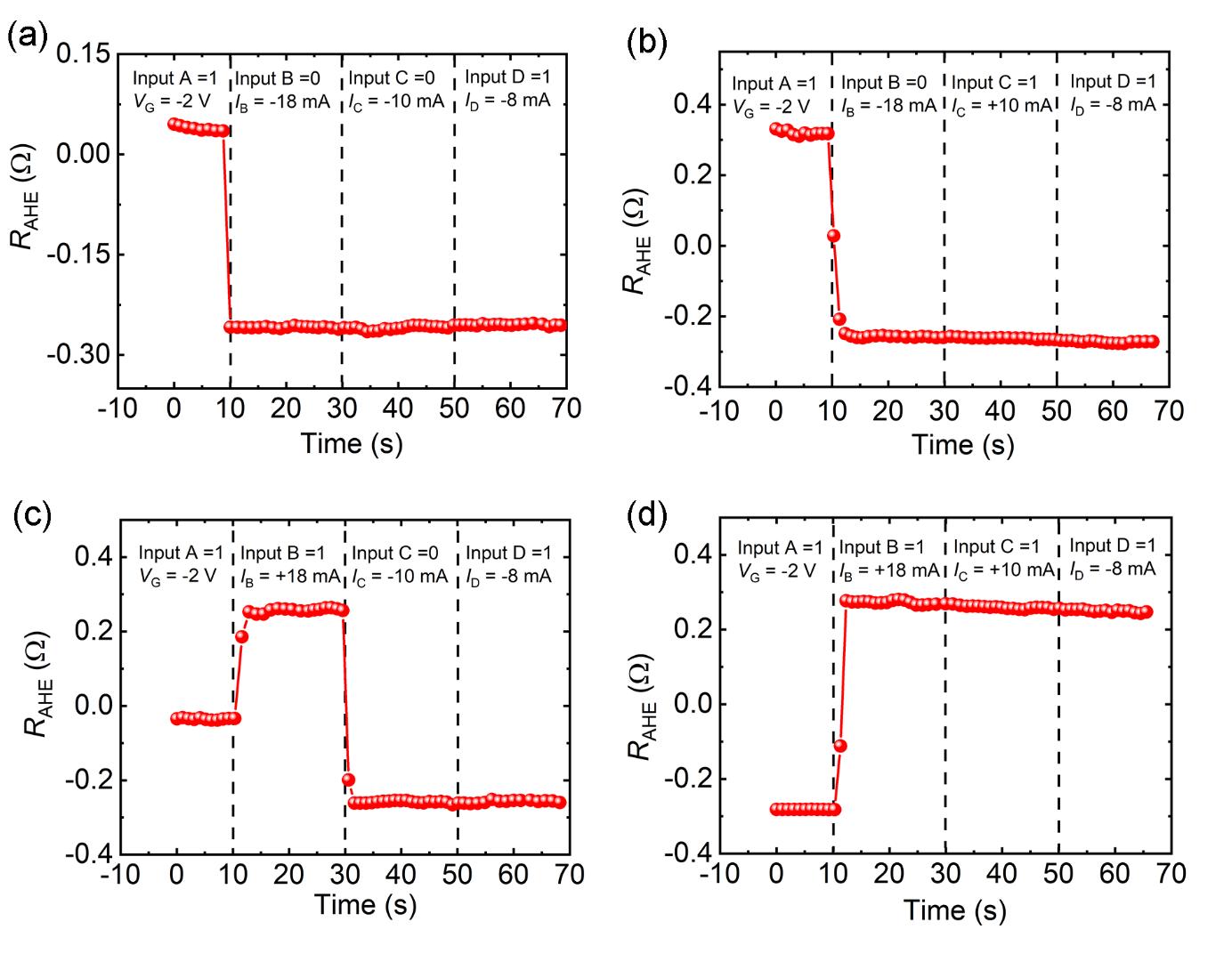


**Figure S23.** The *R*_AHE_ dependent on the time of the logic function for “AND”. a) (1,0,0,1), b) (1,0,1,1), c) (1,1,0,1), d) (1,1,1,1).

Here, we show how to implement all 16 binary Boolean logic operations by applying *V*_G_ and pulse current in TaN(5)/Pt(1.5)/Co(0.9)/Pt(1)/Mn_3_Sn(5)/TaN(1) device. Four logic inputs *V*_G_, *I*_B_, *I*_C_ and *I*_D_ are defined as A, B, C and D, respectively, and the logic output “*L*” is the *R*_AHE_. When *R*_AHE_ > 0, the output is “1”, which for *R*_AHE_ < 0, the output is “0”. The variate A represents the *V*_G_. B, C and D represent the pulse current *I*_B_ *I*_C_ and *I*_D_, respectively. The pulse width is 50 *μ*s, and pulse number is 10. The logic functions are shown in Figure S24 and S25. These logic inputs are completed in three steps. In the first step, *V*_G_ determines the polarity of the SOT-induced magnetization switching (clockwise or anti-clockwise). The second step, the input *I*_B_ initializes the direction of the magnetization. In the third step, the currents *I*_C_ and *I*_D_ as a combined input is used to finalize the magnetization direction. All the 16 binary Boolean logic functions can be realized in TaN(5)/Pt(1.5)/Co(0.9)/Pt(1)/Mn_3_Sn(5)/TaN(1) device.


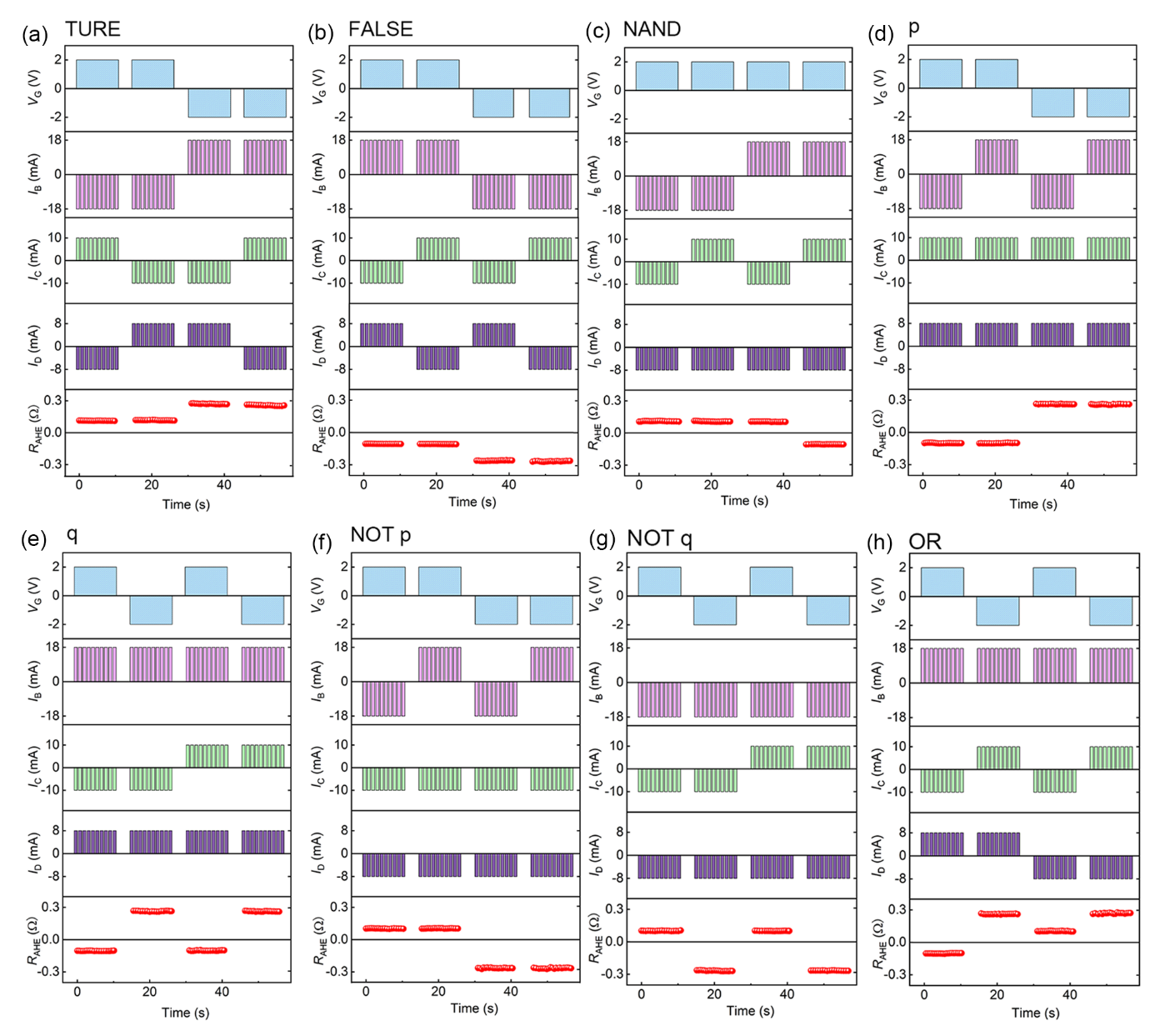


**Figure S24.** Demonstration of Boolean spin logic operations: a) TURE, b) FALSE, c) NAND, d) *p*, e) *q*, f) NOT *p*, g) NOT *q* and h) OR logic functions.


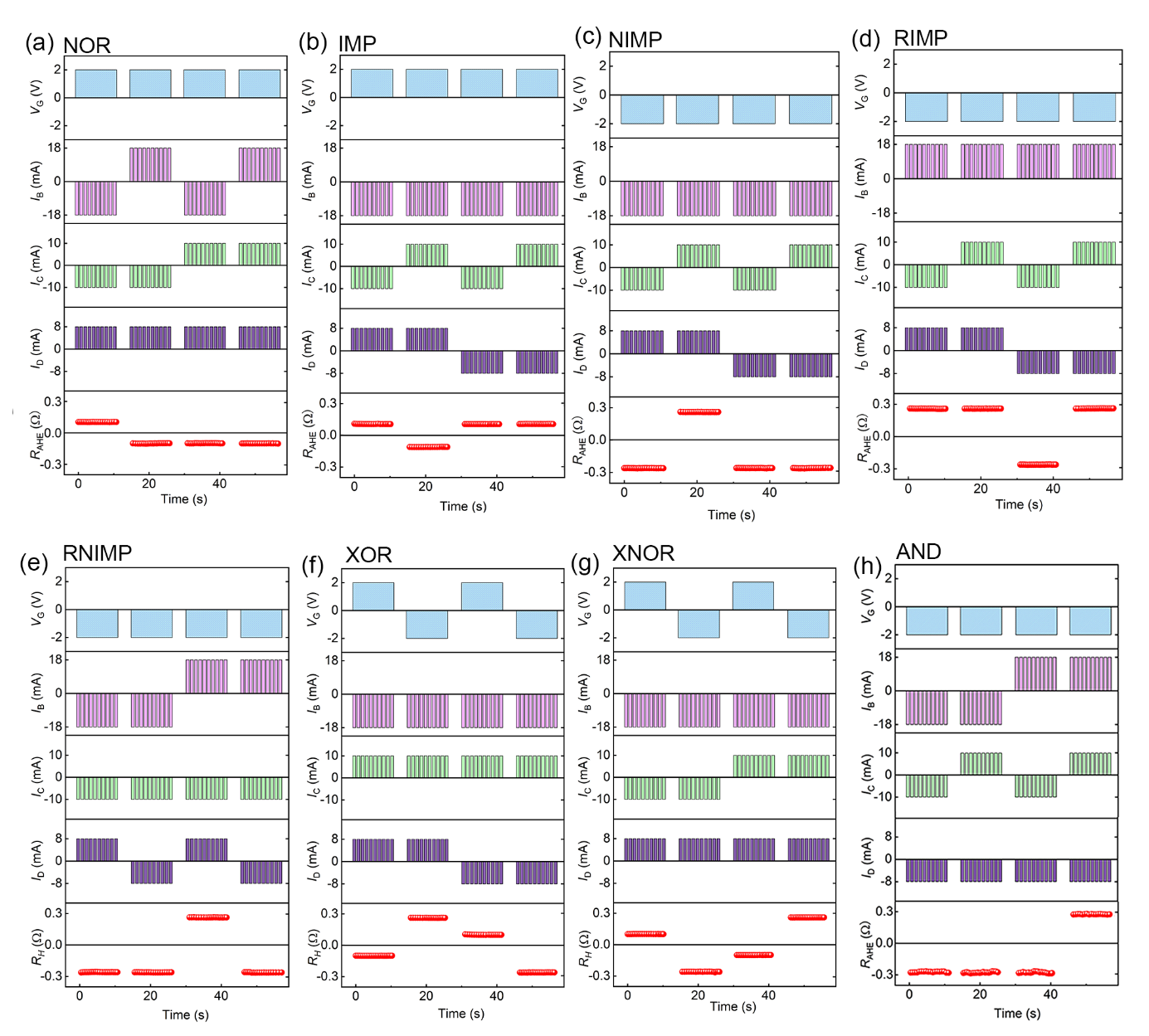


**Figure S25.** Demonstration of Boolean spin logic operation: a) NOR, b) IMP, c) NIMP, d) RIMP, e) RNIMP, f) XOR, g) XNOR and h) AND logic functions.

References

[S1] S. Nakatsuji, N. Kiyohara, T. Higo, *Nature* **2015**, *527*, 212.

[S2] A. Markou, J. M. Taylor, A. Kalache, P. Werner, S. S. P. Parkin, C. Felser, Noncollinear antiferromagnetic Mn3Sn films. *Phys. Rev. Mater.* **2018**, *2*, 051001.

[S3] Y. Kageyama, Y. Tazaki, H. An, T. Harumoto, T. Gao, J. Shi, K. Ando, *Sci. Adv.* **2019**, *5*, eaax4278.

[S4] L. Liu, T. Moriyama, D. C. Ralph, R. A. Buhrman, *Phys. Rev. Lett.* **2011**, *106*, 036601.

[S5] X. Chen, S. Shi, G. Shi, X. Fan, C. Song, X. Zhou, H. Bai, L. Liao, Y. Zhou, H. Zhang, A. Li, Y. Chen, X. Han, S. Jiang, Z. Zhu, H. Wu, X. Wang, D. Xue, H. Yang, F. Pan, *Nat. Mater.* **2021**, *20*, 800.

[S6] T. Nan, C. X. Quintela, J. Irwin, G. Gurung, D. F. Shao, J. Gibbons, N. Campbell, K. Song, S. Y. Choi, L. Guo, R. D. Johnson, P. Manuel, R. V. Chopdekar, I. Hallsteinsen, T. Tybell, P. J. Ryan, J. W. Kim, Y. Choi, P. G. Radaelli, D. C. Ralph, E. Y. Tsymbal, M. S. Rzchowski, C. B. Eom, *Nat. Commun.* **2020**, *11*, 4671.

[S7] D.-H. Kim, M. Haruta, H.-W. Ko, G. Go, H.-J. Park, T. Nishimura, D.-Y. Kim, T. Okuno, Y. Hirata, Y. Futakawa, H. Yoshikawa, W. Ham, S. Kim, H. Kurata, A. Tsukamoto, Y. Shiota, T. Moriyama, S.-B. Choe, K.-J. Lee, T. Ono, *Nat. Mater.* **2019**, *18*, 685.

[S8] S. Kim, P.-H. Jang, D.-H. Kim, M. Ishibashi, T. Taniguchi, T. Moriyama, K.-J. Kim, K.-J. Lee, T. Ono, *Phys. Rev. B* **2017**, *95*, 220402.

[S9] X. Xie, X. Zhao, Y. Dong, X. Qu, K. Zheng, X. Han, X. Han, Y. Fan, L. Bai, Y. Chen, Y. Dai, Y. Tian, S. Yan, *Nat. Commun.* **2021**, *12*, 2473.

[S10] D. Asakura, T. Koide, S. Yamamoto, K. Tsuchiya, T. Shioya, K. Amemiya, V. R. Singh, T. Kataoka, Y. Yamazaki, Y. Sakamoto, A. Fujimori, T. Taira, M. Yamamoto, *Phys. Rev. B* **2010**, *82*, 184419.

[S11] C. T. Chen, Y. U. Idzerda, H. J. Lin, N. V. Smith, G. Meigs, E. Chaban, G. H. Ho, E. Pellegrin, F. Sette, *Phys. Rev. Lett.* **1995**, *75*, 152.

[S12] Y.-W. Oh, S.-h. Chris Baek, Y. M. Kim, H. Y. Lee, K.-D. Lee, C.-G. Yang, E.-S. Park, K.-S. Lee, K.-W. Kim, G. Go, J.-R. Jeong, B.-C. Min, H.-W. Lee, K.-J. Lee, B.-G. Park, *Nat. Nanotechnol.* **2016**, *11*, 878.

[S13] J. Kim, J. Sinha, M. Hayashi, M. Yamanouchi, S. Fukami, T. Suzuki, S. Mitani, H. Ohno, *Nat. Mater.* **2012,** *12*, 240.

[S14] C. O. Avci, K. Garello, M. Gabureac, A. Ghosh, A. Fuhrer, S. F. Alvarado, P. Gambardella, *Phys. Rev. B* **2014**, *90*, 224427.

[S15] R. Chu, L. Liu, B. Cui, W. Liu, T. An, X. Ren, T. Miao, B. Cheng, J. Hu, *ACS Nano* **2022**, *16*, 16077.
